# Efficacy and Outcomes of a Music-Based Emotion Regulation Mobile App in Distressed Young People: Randomized Controlled Trial

**DOI:** 10.2196/11482

**Published:** 2019-01-16

**Authors:** Leanne Hides, Genevieve Dingle, Catherine Quinn, Stoyan R Stoyanov, Oksana Zelenko, Dian Tjondronegoro, Daniel Johnson, Wendell Cockshaw, David J Kavanagh

**Affiliations:** 1 School of Psychology The University of Queensland Brisbane Australia; 2 School of Psychology & Counselling Institute of Health and Biomedical Innovation Queensland University of Technology Brisbane Australia; 3 Centre for Children’s Health Research Queensland University of Technology Brisbane Australia; 4 Creative Industries Faculty Queensland University of Technology Brisbane Australia; 5 School of Business and Tourism Southern Cross University Gold Coast Australia

**Keywords:** depression, anxiety, emotion regulation, music, telehealth, mHealth

## Abstract

**Background:**

Emotion dysregulation increases the risk of depression, anxiety, and substance use disorders. Music can help regulate emotions, and mobile phones provide constant access to it. The *Music eScape* mobile app teaches young people how to identify and manage emotions using music.

**Objective:**

This study aimed to examine the effects of using *Music eScape* on emotion regulation, distress, and well-being at 1, 2, 3, and 6 months. Moderators of outcomes and user ratings of app quality were also examined.

**Methods:**

A randomized controlled trial compared immediate versus 1-month delayed access to *Music eScape* in 169 young people (aged 16 to 25 years) with at least mild levels of mental distress (Kessler 10 score>17).

**Results:**

No significant differences between immediate and delayed groups on emotion regulation, distress, or well-being were found at 1 month. Both groups achieved significant improvements in 5 of the 6 emotion regulation skills, mental distress, and well-being at 2, 3, and 6 months. Unhealthy music use moderated improvements on 3 emotion regulation skills. Users gave the app a high mean quality rating (mean 3.8 [SD 0.6]) out of 5.

**Conclusions:**

Music eScape has the potential to provide a highly accessible way of improving young people’s emotion regulation skills, but further testing is required to determine its efficacy. Targeting unhealthy music use in distressed young people may improve their emotion regulation skills.

**Trial Registration:**

Australian New Zealand Clinical Trials Registry ACTRN12615000051549; https://www.anzctr.org.au/Trial/Registration/TrialReview.aspx?id=365974

## Introduction

### Young People and Emotion Regulation

Mental health and substance use disorders are at their peak and the leading causes of disability and death worldwide in young people (aged 15-25 years) [1]. Anxiety, depression, and substance use disorders are the most common in this age group. Deficits in emotion regulation or the ability to identify, evaluate, express, and modify emotions are important risk and maintaining factors for these disorders [2-5]. Emotion regulation requires the development and integration of several emotion skills such as an awareness of one’s emotional states, directing one’s attention away from the cause of a negative emotion; cognitively reappraising the cause of the negative emotion; and accepting, discharging, or suppressing a negative emotional state [6,7]. Young people commonly experience intense emotional states during puberty and the transition from childhood to adulthood [8]. At the same time, their capacity to fully regulate emotional states is still developing [9,10]. Effective emotion regulation skills may help decrease the intensity and duration of dysphoric states, whereas ineffective emotion regulation may increase them [3].

Only a third of young people with mental disorders seek help [11] and many fail to engage in or complete psychological treatment. Around 60% of young people with anxiety disorders respond to cognitive behavioral therapy, with response rates for unipolar major depression ranging from 48% to 81% over 36 weeks [12-15]. Nevertheless, around 40% of young people with anxiety and 20% to 25% with depressive disorders do not respond to treatment [13,15-17], and 47% of responders to depression treatment relapse within 5 years [18].

### Music and Emotion

Novel interventions targeting young people’s emotion regulation skills could reduce the risk of anxiety or depression disorders and improve help seeking, treatment engagement, and outcomes among those with these disorders. Music listening is one of the favorite leisure activities of young people [19] and one of their most commonly used emotion regulation strategies [20-27], being recently ranked the top stress management strategy in young Australians [28].

Music is commonly used by young people and others to induce, enhance, maintain, or manage moods [29-31]. The impact of music on mood varies according to the goal of the user [32], their pre-existing mood [33], and the type of emotion regulation strategy being used [34]. For example, a correlational study found substantial proportions of adolescents reported improvements in mood when listening to music; these effects were most pronounced when they were happy or bored, rather than angry or sad [33]. Positive effects of music on negative moods appeared constrained by some adolescents preferring angry rather than happy music when in a negative mood [33]. A Web-based experiment found listening to self-selected sad music increased depressive moods and listening to happy music reduced them [34]. However, a partial replication of that study found both participant- and experimenter-selected sad music reduced a depressive mood if a negative mood was induced (via a video clip) before music listening [35]. Finally, music use among people who use cognitive reappraisal as an emotion regulation strategy has been found to enhance well-being, whereas the use of expressive suppression reduced well-being [36]. In summary, music appears to have potential to be used as an effective emotion regulation strategy for improving mood and well-being, although the relationship is complex and controlled studies of adequate power are needed [37].

### Programs Using Music to Target Emotion

The relationship between music and emotions has been harnessed in programs aimed at teaching emotion regulation skills to individuals with mental health problems in clinical and community settings, including eating disorders [38], anxiety disorders [39], substance misuse [40], and schizophrenia [41]. One example is the *Tuned In* group program, which uses hypothetical scenarios and participant-selected music to evoke emotions in sessions to increase emotional awareness and emotion regulation skills. This program demonstrated significant improvements in emotional awareness and regulation post treatment among 41 at-risk adolescents attending an education re-engagement program and 216 adolescents attending an independent mainstream secondary school [42]. A 4-session version of the program among dysphoric first-year university students (n=51; aged 18-25 years) found greater emotional awareness and regulation post treatment compared with a 4-week waitlist control group [43]. These findings provide preliminary evidence that music programs such as *Tuned In* result in positive emotion regulation outcomes.

Media has the potential to enhance the emotion regulation skills and mood of young people in their everyday lives. For instance, a survey study of 229 people found that mood-specific media use might be captured by 3 factors: turning to media in a positive mood, in a negative mood, or in a bored mood [10]. Various forms of difficulty regulating emotion (eg, feeling out of control when upset) predicted media use in negative or bored moods only. More specific analyses show that music use in negative moods is predicted by both positive indices (eg, reflection tendencies) and negative indices of emotion regulation (eg, rumination tendencies), whereas television use in negative moods is only predicted by negative indices of emotion regulation [10]. A systemic review of 23 studies on the use of video games for emotion regulation reported that frequent (but not excessive) video game play, including serious games, may enhance emotion regulation, but commercial gaming offered more opportunities for emotion regulation improvement than limited-time (bespoke) games [44].

Music also has the potential to enhance the emotion regulation skills and mood of young people in their everyday lives. Mobile phones that contain digital music players, personal music libraries, and access to digital radio provide a platform for achieving this. Targeted music apps, therefore, provide an anonymous and highly accessible way of providing young people with the skills to identify, express, and manage emotions in their natural environment [19,45,46]. A recent meta-analysis of 21 studies of electronic health (eHealth) interventions for youth concluded that such apps could result in population-level benefits, even with small effects (*d*=0.13; 95% CI 0.02-0.25) [47].

A growing number of mobile phone apps targeting emotions through music are becoming available. Several approaches are used, including streaming mood-related playlists, providing mood-tagging options for users’ own libraries, and playing sounds and soundscapes to promote relaxation [31]. However, it is unclear how the music mood ratings contained in these are derived, and the majority are not specifically designed to help young people regulate emotions.

### Objectives

This study aimed to evaluate a new app called *Music eScape,* developed to assist young people with identifying, expressing, and managing emotions using music from their own music library. This study reports the 1-month efficacy and 2-, 3-, and 6-month outcomes of the *Music eScape* app in a sample of young people with at least mild mental distress. Potential moderators of app outcomes, including the amount of music use and healthy or unhealthy music use, were examined. In addition, user ratings of the app’s quality were obtained after a month of its use.

## Methods

### Music eScape App

**Figure 1:**
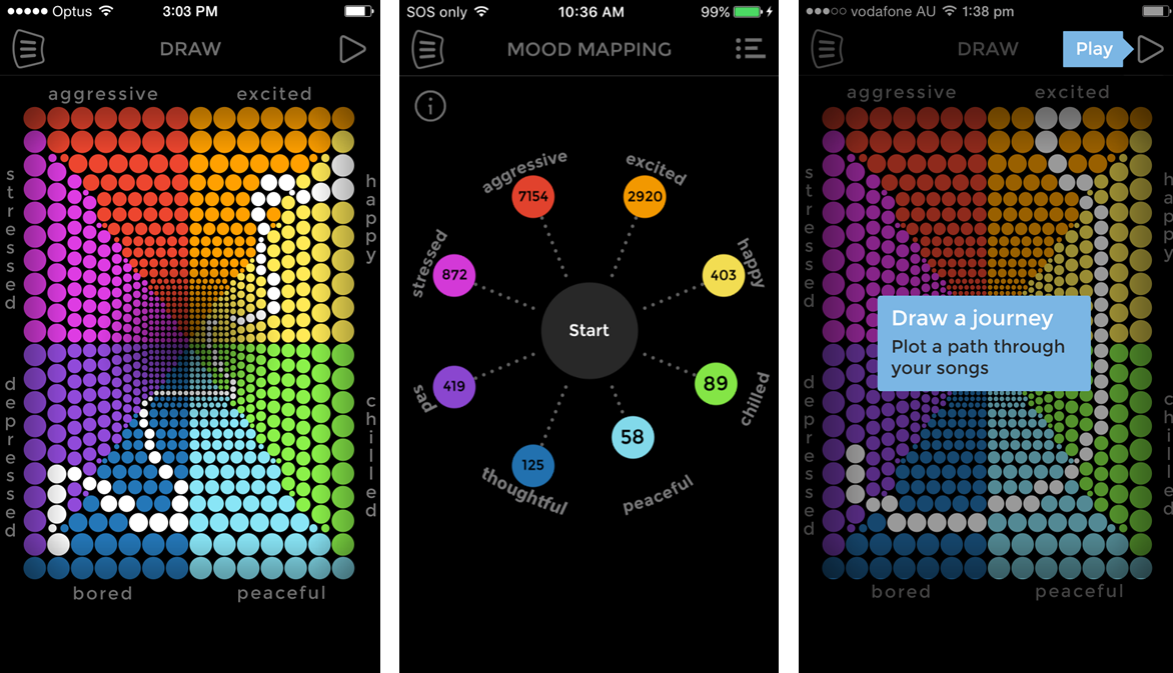
Music eScape app: (left) home screen; (middle) music mood map; and (right) mood journey instructions.

The *Music eScape* app was co-designed by young people and a multidisciplinary research team using a series of participatory design workshops [48]. App design was informed by the dynamic information-motivation-behavioral skills health behavior model [49,50], and agile development processes were used. The *Music eScape* app analyzes each song in the users’ music library according to its level of valence (pleasant to unpleasant) and arousal (very low to very high) using The Echo Nest music data program [51]. The songs are then located in a two-dimensional space consistent with Russell’s circumplex model of emotion [52], labeled around the borders with 8 emotions (see Figure 1, left screenshot): aggressive, excited, happy, chilled, peaceful, bored, depressed, and stressed. Once the music scanning is complete, the user is presented with a mood map of their music (the eScape) to help them identify the prevalent moods of their library (see Figure 1, middle screenshot). Before creating a playlist, the app prompts users to reflect on their current and desired mood and then encourages them to plot a mood journey the playlist will support them to create (see Figure 1, right screenshot). This journey comprises a unique trajectory using their own music (eg, starting in the bored segment and ending in the happy one; see Figure 1, right screenshot). Users can save and label their musical mood journeys (eg, *Chill out*) and can also specify the duration of their playlist, from 15 min to 60 min. They can also select preset mood journeys. After completing their mood journey, users are asked to reflect on their current mood and rate the effectiveness of the playlist they just experienced.

### Participants and Recruitment

Participants were Australian residents aged 16 to 25 years, who reported at least mild distress in the past month on the Kessler 10 Psychological Distress scale (K10>17) and had an iPhone. Recruitment was via student emails and posters in 2 large universities and snowballing techniques. The advertisements invited young people (aged 16-25 years) who owned an iPhone and *felt stressed* to participate in a study testing a new mood management app. They did not include any mention about music in an attempt to avoid recruiting a selective sample of participants with a high affinity to music. The purpose of the study was also concealed during the consent process, such that participants were not aware of the fact that the mood management app used music until they received access to it.

### Measures

#### Emotion Regulation

The 18-item short-form of the Difficulties in Emotion Regulation Scale (DERS-SF) [53] was used to assesses emotion dysregulation on a scale from 1 (almost never) to 5 (almost always). The DERS-SF has excellent reliability and validity and a similar factor structure to the original 36-item scale in adolescents and adults [54]. There are 6 subscales: lack of emotional awareness (alpha=.80), lack of emotional clarity (alpha=.83), difficulties engaging in goal-directed behaviors (alpha=.88), impulse control difficulties (alpha=.91), nonacceptance of emotional responses (alpha=.85), and limited access to emotion regulation strategies (alpha=.85). Participants were also asked to rate their perceived level of success with using music as an emotion regulation strategy on a Likert scale (1=not at all successful to 9=extremely successful; item derived from Thayer et al’s study) [55].

#### Mental Distress and Well-Being

The K10 scale [56] assessed the frequency of psychological distress in the past month, using items rated from 1 (none of the time) to 5 (all of the time). The K10 is a widely used screening tool developed using item response theory to determine the probable presence or absence of a diagnosable anxiety or depressive disorder. Normative data indicate that a cut-off of ≥17 is indicative of at least mild mental distress and is at the seventy-fifth percentile among Australian youth (aged 16-24 years) [57]. Internal consistency was high in this study sample (alpha=.87).

Mental well-being was measured with the Mental Health Continuum-Short Form (MHC-SF) [58,59]. This 14-item scale measures how frequently respondents experienced emotional, psychological, and social well-being in the past month on a 6-point response scale (1=never to 6=every day). The MHC-SF has high levels of reliability and discriminant, convergent, and cross-cultural validity. Internal consistency was .94.

#### Music Measures

A total of 10 items designed specifically for this study explored the level of music education and involvement of participants. Example items include the following: “Do you currently play a musical instrument and/or sing in a group or choir?” (yes or no) and “Do you attend concerts or live music on a regular basis (ie, at least once a month)?” (yes or no).

The Healthy-Unhealthy Music Scale [60] assesses healthy (5 items) and unhealthy (8 items) uses of music, with items rated from 1 (never) to 5 (always). Healthy and unhealthy music use refers to protective (eg, “Music gives me the energy to get going”) versus risky (eg, “When I listen to music I get stuck in bad memories”) forms of music engagement [60]. The healthy subscale has demonstrated concurrent validity with well-being, happiness, and school satisfaction, and its unhealthy subscale is associated with depression, rumination, and stress. Internal consistency in this sample was healthy: alpha=.76 and unhealthy: alpha=.85. A median split was calculated for each of these variables to identify participants scoring either low or high on healthy and low or high on unhealthy music use.

#### App Use and Quality

Backend data on the date, time, frequency, and length of app use were collected. App engagement was defined as the total number of playlists created per participant. App quality was assessed by the Mobile App Rating Scale-User version (uMARS) [61]. This 20-item scale assesses perceived *objective* app quality on 4 subscales (engagement, functionality, aesthetics, and information) rated on a 5-point scale (1=very poor and 5=excellent). Mean subscale scores and a mean objective quality score were derived. Subjective app quality was assessed using 4 questions: “Would you recommend the app?” (1, not to anyone; to 5, everyone); “How many times would you use it?” (1, 0 times; to 5, >50 times); “Would you pay for this app?” (1, no; 2, maybe; and 3, yes); and overall star-rating (1 to 5 stars).

### Procedure

Ethical approval was granted by the relevant university human research ethics committees and the trial was registered with the Australian New Zealand Clinical Trials Registry (ACTRN12615000051549). Informed consent was obtained online before participants completed the baseline online survey. Those meeting study inclusion criteria were automatically identified and randomized via a computerized trial management system to the immediate- or delayed-access groups, with stratification by age group (aged 16-20 years and 21-25 years) and gender.

Those assigned to the immediate-access group were emailed a link to the app. This required users to first download the *TestFlight* app, a beta app distribution platform, which enabled download of the *Music eScape* app before its release in the Apple App Store. Short message service (SMS) text message reminders to access the app were sent at 7-day intervals in the first month.

To minimize attrition, the delayed-access group received 2 SMS text messages during the 1 month wait for access to the app. All baseline and follow-up surveys were completed online. Participants were automatically sent email links to each survey 3 days before, on the day of, and at 3 and 7 days after a follow up was due. Reminder SMS text messages were sent to those who had not completed a follow-up, 8 and 10 days after they were due. Participants were reimbursed Aus $20 for completing each survey.

### Statistical Analyses

The immediate- and delayed-access groups were compared on baseline demographic, mental distress and well-being, emotion regulation, and music variables using logistic regressions, with treatment group allocation as the outcome variable. Data screening indicated all outcomes (ie, emotion regulation, mental distress, and well-being) had acceptable skew and kurtosis. Linear mixed models in SPSS version 25 (IBM Corp, Armonk, NY, USA) were used to conduct intent-to-treat analyses, without prediction of missing data, on the primary outcome variable of difficulties in emotion regulation and secondary outcomes of mental distress and well-being. For all outcomes, time and group main effects and time by group interaction from baseline to 1 month were conducted, followed by analyses examining the impact of the app over time from baseline to the 2-, 3-, and 6-month follow-ups. Gender, baseline duration of music use (hours per week of music listening, with median split into high vs low), and use of music (healthy or unhealthy) were included as control variables and potential moderators of outcomes because of the potential impact of these variables on mood, music, and app use [60]. Two analyses entering app access (yes or no) and app use as additional control variables were also conducted to determine if this varied results. An autoregressive covariance structure (Toeplitz) was specified to account for correlated outcome variables assessed at close time points. Significant effects were probed using pairwise comparisons, and Cohen *d* effect sizes were calculated using SDs pooled across groups and times.

## Results

### Recruitment and Sample Characteristics

**Figure 2:**
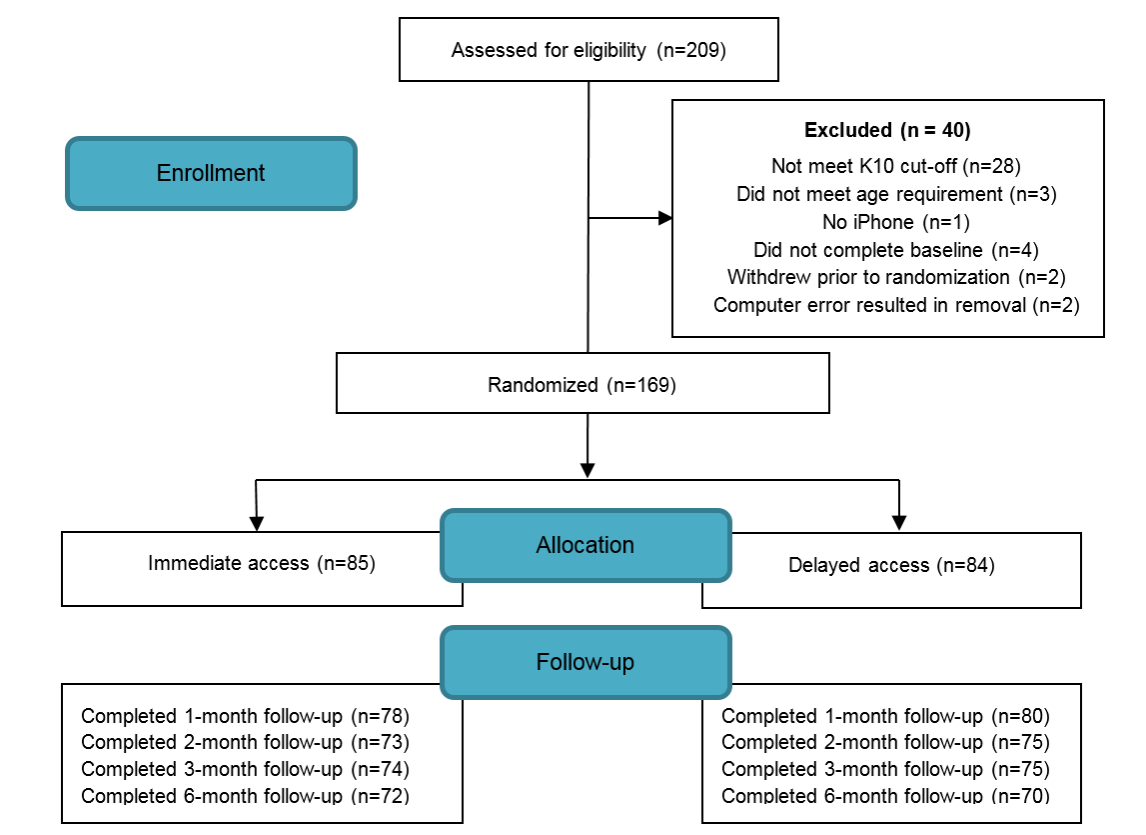
Consort diagram.

Figure 2 displays the consort diagram. A total of 209 young people responded to recruitment advertisements and completed the online survey. Of those, 80.9% (169/209) met full study inclusion criteria and were allocated to immediate (n=85) or delayed (n=84) app access. Follow-up rates were high (93.5% at 1 month, 87.6% at 2 months, 88.2% at 3 months, and 84.0% at 6 months). There was no significant difference in key demographic factors (eg, age, gender, work status, and education) between those who completed all follow-up surveys and those who missed 1 or more postbaseline assessments.

Demographic characteristics of the sample are displayed in Table 1, and descriptive statistics for the primary and secondary outcome variables are provided in Table 2. There were no significant differences between immediate and delayed groups on any baseline demographic, music, or primary or secondary outcome variables.

**Table 1:** Demographic and baseline music characteristics.

Characteristics				Total (N=169)	Immediate (N=85)	Delayed (N=84)	*P* value
**Demographics**							
	Age, mean (SD)			19.9 (2.5)	20.0 (2.6)	19.9 (2.4)	.9
	Gender (females), n (%)			134 (79.3)	67 (78.8)	67 (79.8)	.82
	English language fluency, n (%)			139 (82.2)	68 (80.0)	71 (84.5)	.44
	Aboriginal or Torres Strait Islander, n (%)			2 (1.2)	1 (1.2)	1 (1.2)	.99
**Education, n (%)**							.29
	Degree, postgraduate study			40 (23.7)	17 (19.0)	23 (27.4)	
	Certificate or diploma			25 (14.8)	13 (15.3)	12 (14.3)	
	High school			104 (61.6)	55 (64.7)	49 (58.4)	
**Work status, n (%)**							.39
	Full-time			16 (9.5)	6 (7.1)	10 (11.9)	
	Part-time or casual			43 (25.4)	28 (32.9)	15 (17.9)	
	Unemployed or disability allowance			10 (5.9)	4 (4.7)	6 (7.1)	
	Home duties			1 (0.6)	0 (0.0)	1 (1.2)	
	Volunteer work			2 (1.2)	1 (1.2)	1 (1.2)	
	Full-time or part-time student			97 (57.4)	46 (54.1)	51 (60.7)	
**Relationship status, n (%)**							.06
	Married			3 (1.8)	2 (1.8)	1 (1.2)	
	In a relationship			85 (50.3)	50 (40.6)	35 (41.7)	
	Single			81 (47.9)	33 (38.8)	48 (57.1)	
Current psychological treatment, n (%)				38 (22.5)	17 (20.0)	21 (25.0)	.44
Use smartphone daily, n (%)				155 (91.7)	76 (89.4)	79 (94.0)	.94
**Music, n (%)**							
	Accessed music online in past month			164 (97.0)	82 (96.5)	82 (97.6)	.66
	**Play musical instrument or sing in choir**						
			Past	139 (82.2)	71 (83.5)	68 (81.0)	.9
			Current	47 (27.8)	24 (28.2)	23 (27.4)	.66
	Compose music			52 (30.8)	27 (31.8)	25 (29.8)	.78
	Attend concerts or live music at least monthly			50 (29.6)	29 (34.1)	21 (25.0)	.2
**Music listening each week, mean (SD)**							
		Average number of hours per day		2.6 (2.3)	2.8 (2.7)	2.5 (1.9)	.31
		Average number of days		6.2 (1.6)	6.3 (1.6)	6.1 (1.5)	.6
		Median split (days×hours)—High		98 (46.9)	43 (51.2)	48 (57.1)	.39
		**HUMS^a^**					
			High healthy music use	128 (61.2)	61 (71.8)	53 (63.1)	.23
			High unhealthy music use	97 (46.4)	41 (48.2)	49 (58.3)	.19
At least moderate success at listening to music to change mood, n (%)				167 (87.9)	75 (88.2)	73 (86.9)	.66

^a^HUMS: Healthy Unhealthy Music scale.

**Table 2:** Means and SDs for all primary and secondary outcome variables.

Measure	Group	Baseline, mean (SD)	1 month, mean (SD)	2 month, mean (SD)	3 month, mean (SD)	6 month, mean (SD)
DERS^a^ aware	Immediate	7.82 (2.66)	8.01 (2.67)	8.10 (2.68)	8.07 (2.74)	7.75 (2.73)
	Delayed	7.49 (2.21)	8.03 (2.65)	8.05 (2.66)	7.77 (2.79)	7.49 (2.73)
	Total	7.66 (2.45)	8.02 (2.65)	8.07 (2.66)	7.92 (2.76)	7.62 (2.72)
Clarity	Immediate	7.51 (2.68)	6.88 (2.40)	6.86 (2.39)	6.80 (2.50)	6.49 (2.68)
	Delayed	7.95 (2.64)	6.98 (2.62)	7.23 (2.64)	6.51 (2.43)	7.20 (2.61)
	Total	7.73 (2.66)	6.93 (2.51)	7.05 (2.52)	6.65 (2.47)	6.84 (2.66)
Goals	Immediate	10.62 (2.99)	9.65 (2.74)	8.84 (3.05)	8.69 (3.23)	8.99 (3.05)
	Delayed	11.17 (2.96)	10.03 (3.06)	9.58 (2.87)	9.49 (3.06)	9.21 (2.67)
	Total	10.89 (2.98)	9.84 (2.91)	9.21 (2.97)	9.09 (3.16)	9.10 (2.86)
Impulse	Immediate	6.69 (3.07)	6.11 (2.69)	5.81 (2.60)	5.53 (2.48)	5.72 (2.41)
	Delayed	7.13 (3.38)	6.36 (2.80)	6.01 (2.68)	6.03 (2.82)	6.10 (2.51)
	Total	6.91 (3.23)	6.24 (2.74)	5.91 (2.64)	5.78 (2.66)	5.91 (2.46)
Nonacceptance	Immediate	8.24 (3.19)	7.32 (2.83)	7.11 (2.48)	7.05 (2.74)	7.18 (3.01
	Delayed	8.35 (3.24)	7.36 (2.83)	7.66 (3.33)	7.56 (3.01)	7.60 (3.08)
	Total	8.29 (3.21)	7.34 (2.82)	7.39 (2.94)	7.31 (2.88)	7.39 (3.04)
Strategies	Immediate	7.47 (3.28)	7.06 (2.84)	6.33 (2.63)	6.36 (2.71)	6.64 (2.71)
	Delayed	7.89 (3.40)	7.00 (2.89)	6.89 (2.77)	6.75 (3.18)	6.33 (2.67)
	Total	7.68 (3.34)	7.03 (2.86)	7.92 (2.71)	6.55 (2.95)	6.49 (2.69)
Kessler 10	Immediate	27.52 (6.91)	23.00 (6.47)	22.51 (6.92)	22.83 (7.55)	22.68 (7.97)
	Delayed	28.33 (6.59)	24.19 (6.94)	22.41 (6.50)	21.96 (6.93)	22.46 (7.55)
	Total	27.92 (6.74)	23.60 (6.72)	22.36 (6.69)	22.39 (7.24)	22.57 (7.74)
MHC-SF^b^	Immediate	52.53 (12.72)	55.85 (12.76)	55.25 (13.81)	57.61 (14.80)	59.88 (13.87)
	Delayed	50.64 (14.88)	52.34 (14.89)	53.08 (15.02)	54.97 (15.32)	54.14 (14.62)
	Total	51.59 (13.82)	54.07 (13.94)	54.16 (14.42)	56.29 (15.07)	57.05 (14.49)

^a^DERS: Difficulties in Emotion Regulation scale.

^b^MHC-SF: Mental Health Continuum-Short Form.

### App Use and Quality

Backend data indicated that 12 participants did not download the app, a further 34 downloaded but never used the app, 31 downloaded it but experienced technical flaws, and 7 were allocated to the immediate condition but did not download the app until a month after allocation (included in this group for intent-to-treat purposes). Of those who downloaded the app, the total number of generated playlists ranged from 0 to 71, (median=2). No playlists were generated by 41%, and only 7.5% of the sample generated more than 15 playlists. The number of generated playlists did not vary significantly between immediate- and delayed-access groups or by gender. The duration of app music use variable was considered unreliable as it was not possible to gauge the extent to which participants were listening to the music (vs leaving the app open with music playing).

On the uMARS, the app had a high level of objective app quality (mean_overall_*=* 3.8 [SD 0.50]), with good engagement (mean 3.67 [SD 0.61]), aesthetics (mean 4.10 [SD 0.63]), and information (mean 4.05 [SD 0.61]), and acceptable functionality (mean 3.47 [SD 0.66]). Participants reported they would use the app between 10 and 50 times (mean 4.09 [SD 1.04]), and although they were unlikely to pay for the app (mean 2.43 [SD 1.23]), they gave it a 3.6 out of 5-star rating (SD 0.65).

### Emotion Regulation Outcomes

The linear mixed model revealed no time by group interaction for any of the 6 difficulties in emotion regulation subscales of the DERS (see Table 3). Time effects were found on 5 of the 6 DERS subscales (clarity, goals, nonacceptance, strategies, and impulse) when comparing baseline both with the 1-month follow-up and with the 2-, 3-, and 6-month follow-ups (see Table 3). These effects did not vary when controlling for whether participants used the app (yes or no) or the level of app use.

**Figure 3:**
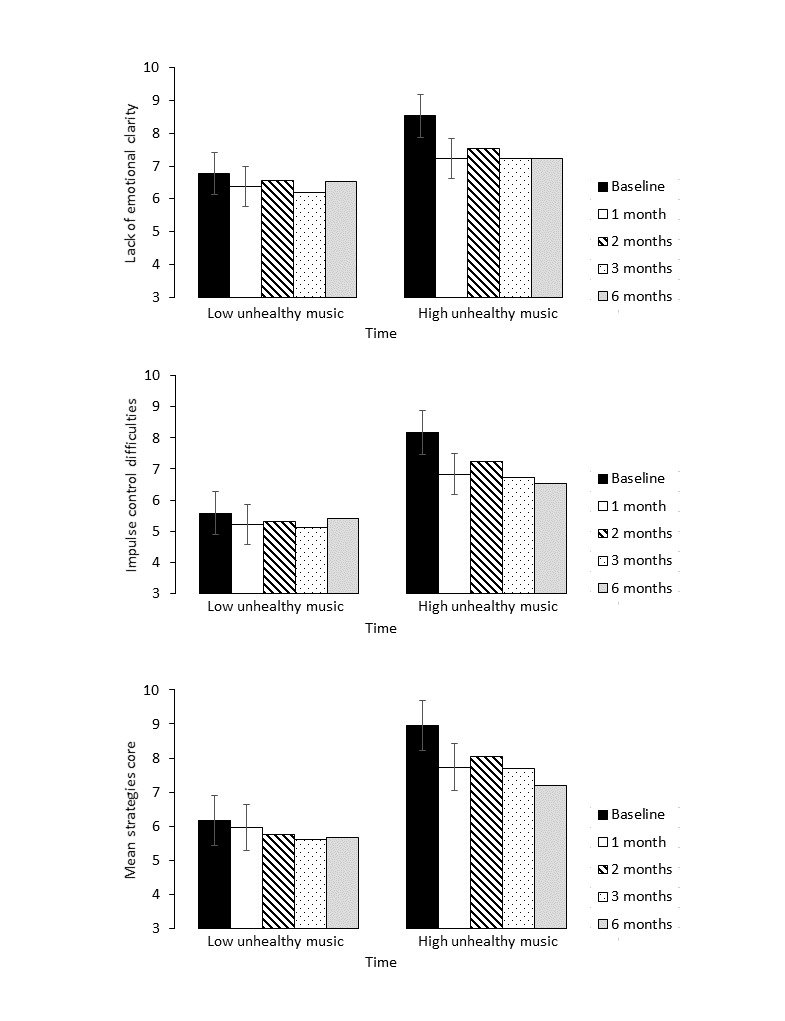
Adjusted mean emotion regulation (difficulties in emotional regulation scale) scores and unhealthy use of music.

To better understand these changes over time, moderating effects of gender, duration of music use, and healthy or unhealthy music use were assessed across all time points. For difficulties engaging in goal-directed behavior when distressed (DERS-Goal) and nonacceptance of emotional responses (DERS-nonacceptance), no significant moderating effects were found for gender (*F*_4,328_=2.12; *P*=.07; *F*_4,351_=1.16; *P*=.33), duration of music use (*F*_4,361_=1.00; *P*=.40; *F*_4,366_=0.32; *P*=.87), unhealthy use of music (*F*_4,369_=0.53; *P*=.72; *F*_4,373_=1.98; *P*=.09), or healthy use of music (*F*_4,363_=1.63; *P*=.17; *F*_4,355_=0.20; *P*=.94). When exploring the time main effects, difficulties engaging in goal-directed behavior decreased from baseline to 1 month (mean_difference_=−1.04, 95% CI −1.44 to −0.65; *t*_383_=5.24; *P*<.001; *d*=0.36) and from 1 to 2 months (mean_difference_=−0.59, 95% CI −1.03 to −0.14; *t*_383_=2.59; *P*=.01; *d*=0.22), before maintaining stability at 3 and 6 months (*P*=.72; *P*=.80; see Figure 3). Nonacceptance of emotional responses decreased from baseline to 1 month (mean_difference_=−0.59, 95% CI −1.41 to −0.55; *t*_376_=4.49; *P*<.001; *d*=0.32) and maintained stability thereafter (*P*=.46; *P*=.81; *P*=.82).

**Table 3:** Emotion regulation, mental distress, and well-being outcomes.

Measure	Comparison	Main effects	*F* test *(df)*	*P* value* *
DERS^a^ awareness	Baseline vs 1 month	Time	3.62 (1,157)	.06
		Time×group	0.66 (1,157)	.42
	Baseline vs 2, 3, and 6 months	Time	1.52 (3,252)	.22
		Time×group	0.18 (3,252)	.91
Clarity	Baseline vs 1 month	Time	19.70 (1,158)	<.001
		Time×group	1.34 (1,158)	.25
	Baseline vs 2, 3, and 6 months	Time	11.66 (3,300)	<.001
		Time×group	1.67 (3,300)	.17
Goals	Baseline vs 1 month	Time	25.73 (1,157)	<.001
		Time×group	0.46 (1,157)	.5
	Baseline vs 2, 3, and 6 months	Time	31.76 (3,325)	<.001
		Time×group	1.21 (3,325)	.31
Impulse	Baseline vs 1 month	Time	9.90 (1,154)	.002
		Time×group	0.73 (1,154)	.39
	Baseline vs 2, 3, and 6 months	Time	10.56 (3,307)	<.001
		Time×group	0.20 (3,307)	.89
Nonacceptance	Baseline vs 1 month	Time	19.99 (1,156)	<.001
		Time×group	0.00 (1,156)	.96
	Baseline vs 2, 3, and 6 months	Time	8.18 (3,366)	<.001
		Time×group	0.67 (3,366)	.57
Strategies	Baseline vs 1 month	Time	11.50 (1,155)	<.001
		Time×group	1.85 (1,155)	.18
	Baseline vs 2, 3, and 6 months	Time	12.53 (3,297)	<.001
		Time×group	1.92 (3,297)	.13
K10^b^	Baseline vs 1 month	Time	74.77 (1,159)	<.001
		Time×group	0.02 (1,159)	.9
	Baseline vs 2, 3, and 6 months	Time	48.11 (3,248)	<.001
		Time×group	0.77 (3,248)	.51
MHC-SF^c^	Baseline vs 1 month	Time	9.60 (3,155)	.002
		Time×group	0.70 (1,155)	.4
	Baseline vs 2, 3, and 6 months	Time	12.75 (3,260)	<.001
		Time×group	1.43 (3,260)	.24

^a^DERS: Difficulties in Emotion Regulation Scale.

^b^K10: Kessler 10.

^c^MHC-SF: Mental Health Continuum-Short Form.

Moderating effects were found for unhealthy use of music, for lack of emotional clarity (*F*_4,394_=2.43; *P*=.05; Figure 3), impulse control difficulties (*F*_4,316_=3.18; *P*=.01; Figure 3), and limited access to emotion regulation strategies (*F*_4,443_=2.78; *P*=.03; Figure 3), but not for healthy use of music (*F*_4,387_=2.06; *P*=.09; *F*_4,310_=2.36; *P*=.05; *F*_4,444_=0.35; *P*=.84). No moderating effects were found for gender (*F*_4,158_=0.02; *P*=.98; *F*_4,295_=1.89; *P*=.11; *F*_4,425_=1.51; *P*=.20) or duration of music use (*F*_4,387_=1.86; *P*=.12; *F*_4,315_=0.41; *P*=.80; *F*_4,441_=0.52; *P*=.72).

Post hoc pairwise comparisons with a Bonferroni adjusted *P* value <.01 revealed an adjusted mean decrease in difficulties with emotional clarity from baseline to 1 month follow-up for those who reported high use of unhealthy music (mean_difference_=−1.29, 95% CI −1.83 to −0.75; *t*_387_=4.72; *P*<.001; *d*=0.43) but not low (mean_difference_=−0.40, 95% CI −0.98 to 0.18; *t*_387_=1.35; *P*=.18; *d*=0.16; Figure 3). Similar results were found for impulse control difficulties (high unhealthy music: mean_difference_=−1.34, 95% CI −1.94 to −0.75; *t*_310_=4.45; *P*<.001; *d*=0.34 and low unhealthy music: mean_difference_=−0.09, 95% CI −0.81 to 0.63; *t*_310_=0.23; *P*=.81; *d*=0.07; Figure 3) and for limited access to emotion regulation strategies (high unhealthy music: mean_difference_=−1.23, 95% CI −1.84 to −0.63; *t*_443_=4.01; *P*<.001; *d*=0.35 and low unhealthy music: mean_difference_=0.20, 95% CI −0.52 to 0.91; *t*_443_=0.54; *P*=.59; *d*=0.03; Figure 3). For all 3 emotion regulation variables, reductions were stable at all subsequent time points (Figure 3).

### Mental Distress and Well-Being Outcomes

The linear mixed model revealed time main effects but no time by group interaction for mental distress (K10) or well-being (MHC-SF; Table 3). These effects did not vary when controlling for app access (yes or no) or use.

To better understand these changes over time, moderating effects of gender, duration of music use, and unhealthy or healthy music use were assessed across all time points. For mental distress, moderating effects were found for gender (*F*_4,254_=3.09; *P*=.02) but not for duration of music use (*F*_4,272_=0.74; *P*=.57) or for healthy (*F*_4,266_=1.70; *P*=.15) or unhealthy (*F*_4,272_=0.77; *P*=.55) use of music. Post hoc pairwise comparisons, with a Bonferroni adjusted *P* value of .003, revealed an adjusted mean decrease in mental distress from baseline to 1 month for females (mean_difference_=−4.50, 95% CI −5.66 to −3.34; *t*_272_=7.68; *P*<.001; *d*=0.37) but not for males (mean_difference_=−0.12, 95% CI −2.07 to 2.31; *t*_272_=0.11; *P*=.92; *d*=0.71).

For well-being, no significant moderating effects were found for gender (*F*_4,283_=1.55; *P*=.19), duration of music use (*F*_4,308_=2.41; *P*=.05), unhealthy use of music (*F*_4,310_=0.13; *P*=.97), or healthy use of music (*F*_4,301_=0.42; *P*=.79). When exploring the time main effects, there was no change in well-being scores when comparing baseline with 1-month (*P*=.10) or 2-month assessments (*P*=.40). However, there was a significant increase in well-being from baseline to 3 months (mean_difference_=3.09, 95% CI 0.88-5.29; *t*_278_=2.76; *P*=.006; *d*=0.33), which was then maintained at the 6 months (3 vs 6 month: *P*=.58).

## Discussion

### Principal Findings

This study examined the 1-month efficacy and 2-, 3-, and 6-month outcomes of the *Music eScape* app in 169 young people with at least mild levels of mental distress. The trial found no differential improvements from app access at 1 month in emotion regulation, mental distress, or well-being. Nevertheless, improvements on 5 out of the 6 emotion regulation strategies, mental distress, and well-being were evident in both groups over the 6-month trial.

The lack of significant differences between the immediate versus 1-month delayed-access groups indicates that the *Music eScape* app was ineffective in achieving change in emotion regulation, mental distress, or well-being, beyond the impact of research assessments alone. Gender, duration of music use, unhealthy and healthy music use, and app use did not impact these results. Although the use of a 1-month delayed-access control may have limited our ability to find effects, waitlist control conditions are commonly used in mobile health (mHealth) research. For example, 2 recent meta-analyses of smartphone mental health apps reporting 8 out of 18 studies on depression and 4 out of 9 studies on anxiety used waitlist controls [62,63]. The duration of the delay ranged from 4 to 16 weeks, depending on the length of the mHealth intervention [62,63]. Although the meta-analyses found mHealth apps had small to moderate effects on both depression and anxiety outcomes, 2 out of the 3 included studies that used a 1-month waitlist control found no effects [57,58]. Thus, the 1-month delay used in this trial might have been insufficient for participants to receive an adequate dose of the *Music eScape* app. Baseline data also indicated that participants had high levels of music use (2.6 hours per day) and emotional awareness (DERS subscale), and 88% participants reported at least moderate levels of success using music to change their mood, suggesting a ceiling effect may have been present on these variables. Nevertheless, improvements in emotion regulation were found on the 5 DERS subscales across the whole sample, suggesting that this study had the ability to detect a change in emotion regulation across time.

Both groups had access to the *Music eScape* app after the first month. Although improvements in mental distress, well-being, and emotion regulation were found over the 6 months, it is not possible to attribute these results to the app. These improvements may have been because of regression to the mean or assessment effects, particularly given that the 5 assessments were completed over a 6-month period. The amount of app use did not affect any outcomes.

To better understand the changes in emotion regulation strategies, over time, moderating effects of gender, duration of music use, and healthy or unhealthy music use were explored. Results indicated that improvements in emotional clarity, impulse control, and limited access to emotion regulation strategies were only found in distressed young people who engaged in high levels of unhealthy music use at baseline. This finding highlights the potential importance of targeting unhealthy music use to improve the emotion regulation skills of distressed young people. Currently, the app allows users to maintain or intensify their current mood by choosing mood-congruent music. The moderation of outcomes by the degree of unhealthy use of music suggests that some may have used the app to stay in a negative mood, consistent with observations in previous research [30-32]. According to meta-emotion theories, some people are drawn toward emotional experiences, whereas others are motivated to avoid and control emotional experiences. Bartsch et al [44] draw links between these individual meta-emotional tendencies and selective of media use such as watching melodramatic or horror movies (which some people enjoy, whereas others prefer to avoid). Similarly, some people enjoy listening to sad and angry music [64], and there is some evidence that this music-induced emotional exposure is related to better emotional processing and well-being [65-67]. Other research suggests that immersion in sad and angry music might be unhealthy for some young people who are prone to depression or other mental health problems [37]. Further research is required to determine whether the *Music eScape* app can improve emotion regulation skills through reductions in unhealthy music use, particularly if coaching is provided on its use for that purpose. Prospective research is also required to determine whether unhealthy music use is a correlate or risk factor for depression in young people and whether it moderates emotion regulation skills in young people without depression.

No moderators of well-being outcomes across the 6 months were found, and only female gender, but not the amount or type (healthy or unhealthy) of music use, moderated improvements in mental distress. The reduction in distress for female but not male participants may be partly because of increased power in detecting changes for females, given that 79% of the sample was female. However, no gender differences in app use were found, and gender has not been found to moderate depression or anxiety outcomes in systematic reviews of psychological treatment trials [68,69]. Further research is required to determine if gender moderates eHealth treatment outcomes.

On average, young people gave *Music eScape* a 4 out of 5 rating for overall objective app quality and for information and aesthetics scales and 3 out of 5 for engagement and functionality. These uMARS scores were higher than recent MARS expert ratings on 50 other eHealth apps [61]. Young people reported they would use the app 10 to 50 times and generated a median of 2 playlists, which is sufficient for users to learn how to identify and manage their mood using music. However, 7.1% (12/169) participants did not download the app, 20.1% (34/169) participants downloaded but did not use the app, and a further 18.3% (31/169) participants experienced technical difficulties using the app. Although the level of uptake or usage of music intervention apps is unknown, 2018 Localytics data on 37,000 apps indicate 21% apps are used only once, with use of 62% of apps discontinued before the eleventh use [70]. In comparison, the current data suggest a high level of maintained use.

### Strengths and Limitations

A large community sample of 169 young people with at least mild distress (K10 scores of >17) participated in this trial. However, the volunteer sampling method used to recruit participants limits the generalizability of results. Despite efforts to avoid participants with an affinity for music during recruitment, the sample used music for substantial average durations at baseline (2+ hours per 6 days a week). Many also used music for emotion regulation strategy, which may have created ceiling effects on key outcome variables. We were also only able to use the number of app playlists generated as an indicator of app use rather than the duration of app music use.

Strengths of this study include its high participation (80%), app uptake (91.5%), and retention rates in research follow-ups (87%-96%) and the inclusion of a range of potential control variables and moderators of outcomes. However, there might have been other unmeasured moderators that future research may identify. Assessment reactivity could be reduced by minimizing the length of research assessments and masking participants from the research hypotheses (eg, comparing music apps with other health apps that collect information on emotions) and assessment process (eg, including questions on emotions as part of a general health survey).

The music available through the current version of *Music eScape* is limited to the users’ own music library. However, 97% of the sample reported accessing music online in the past week. Future versions of the app, which interface with music streaming services that give users access to a much wider repertoire, may enhance its effects by giving users a choice of preferred music for each mood journey step or ensuring that different (or more current) music is offered each time they use the app.

Further testing is required to demonstrate whether the app has effects on emotion regulation, mental health, and well-being over a longer delayed-access period, and if so, whether it has superior effects compared with placebo control apps or other emotion regulation apps or interventions. Additional benefits from adding the app to other interventions for emotion regulation in young people could also be tested.

### Conclusions

Mobile app use is increasingly prevalent worldwide, particularly among young people who are also the biggest consumers of music. The *Music eScape* app is freely available to young people, parents, and practitioners via the Google Play store. Unfortunately, since this study has completed, iOS updates have led to the app being currently unavailable via the Apple App Store. Although further testing is required to demonstrate efficacy, the results of this study highlight the potential of music intervention apps such as *Music eScape* to deliver engaging and highly accessible emotion regulation skills training to young people in real time in their natural environment, which in principle could result in population-wide benefits in mental distress and well-being.

## Abbreviations

CRC: Cooperative Research Centre
DERS: Difficulties in Emotion Regulation Scale
DERS-SF: Difficulties in Emotion Regulation Scale-Short Form
eHealth: electronic health
K10: Kessler 10 Psychological Distress scale
MHC-SF: Mental Health Continuum-Short Form
mHealth: mobile health
SMS: short message service
uMARS: Mobile App Rating Scale-User Version

## Appendix

Multimedia Appendix 1 CONSORT-EHEALTH checklist (V 1.6.1).
