# Supplementary material for: Efficacy and Outcomes of a Music-Based Emotion Regulation Mobile App in Distressed Young People: Randomized Controlled Trial
Source: JMIR Mhealth Uhealth. 2019 Jan 16;7(1):e11482. doi: 10.2196/11482 (PMC6352004; doi:10.2196/11482)
Supplement: Multimedia Appendix 1 [file mhealth_v7i1e11482_app1.pdf]

CONSORT-EHEALTH (V 1.6.1) - Submission/Publication Form

The CONSORT-EHEALTH checklist is intended for authors of randomized trials evaluating web-based and Internet-based applications/interventions, including mobile interventions, electronic games (incl multiplayer games), social media, certain telehealth applications, and other interactive and/or networked electronic applications. Some of the items (e.g. all subitems under item 5 - description of the intervention) may also be applicable for other study designs.

The goal of the CONSORT EHEALTH checklist and guideline is to be
a) a guide for reporting for authors of RCTs,
b) to form a basis for appraisal of an ehealth trial (in terms of validity)

CONSORT-EHEALTH items/subitems are MANDATORY reporting items for studies published in the Journal of Medical Internet Research and other journals / scientific societies endorsing the checklist.

Items numbered 1., 2., 3., 4a., 4b etc are original CONSORT or CONSORT-NPT (non-pharmacologic treatment) items.
Items with Roman numerals (i., ii, iii, iv etc.) are CONSORT-EHEALTH extensions/clarifications.

As the CONSORT-EHEALTH checklist is still considered in a formative stage, we would ask that you also RATE ON A SCALE OF 1-5 how important/useful you feel each item is FOR THE PURPOSE OF THE CHECKLIST and reporting guideline (optional).

Mandatory reporting items are marked with a red *.
In the textboxes, either copy & paste the relevant sections from your manuscript into this form - please include any quotes from your manuscript in QUOTATION MARKS,
or answer directly by providing additional information not in the manuscript, or elaborating on why the item was not relevant for this study.

YOUR ANSWERS WILL BE PUBLISHED AS A SUPPLEMENTARY FILE TO YOUR PUBLICATION IN JMIR AND ARE CONSIDERED PART OF YOUR PUBLICATION (IF ACCEPTED).
Please fill in these questions diligently. Information will not be copyedited, so please use proper spelling and grammar, use correct capitalization, and avoid abbreviations.

DO NOT FORGET TO SAVE AS PDF _AND_ CLICK THE SUBMIT BUTTON SO YOUR ANSWERS ARE IN OUR DATABASE !!!

Citation Suggestion (if you append the pdf as Appendix we suggest to cite this paper in the caption):
Eysenbach G, CONSORT-EHEALTH Group
CONSORT-EHEALTH: Improving and Standardizing Evaluation Reports of Web-based and Mobile Health Interventions
J Med Internet Res 2011;13(4):e126
URL: http://www.jmir.org/2011/4/e126/
doi: 10.2196/jmir.1923
PMID: 22209829

* Required

Your name *

First Last

Stoyan Stoyanov

Primary Affiliation (short), City, Country *

University of Toronto, Toronto, Canada

Queensland University of Technology

Your e-mail address *

abc@gmail.com

stoyan.stoyanov@qut.edu.au

Title of your manuscript *

Provide the (draft) title of your manuscript.

Efficacy and Outcomes of a Music-Based Emotion Regulation Mobile App in Distressed Young People: Randomized Controlled Trial

Name of your App/Software/Intervention *

If there is a short and a long/alternate name, write the short name first and add the long name in brackets.

Music eScape

Evaluated Version (if any)

e.g. "V1", "Release 2017-03-01", "Version 2.0.27913"

Your answer

Language(s) *

What language is the intervention/app in? If multiple languages are available, separate by comma (e.g. "English, French")

English

URL of your Intervention Website or App

e.g. a direct link to the mobile app on app in appstore (itunes, Google Play), or URL of the website. If the intervention is a DVD or hardware, you can also link to an Amazon page.

https://apkpure.com/music-escape-unre

URL of an image/screenshot (optional)

Your answer

Accessibility *

Can an enduser access the intervention presently?

- ○ access is free and open
- ○ access only for special usergroups, not open
- ○ access is open to everyone, but requires payment/subscription/in-app purchases
- ○ app/intervention no longer accessible
- ◉ Other: App no longer accessible on Apple App Store. A Beta version availab

Primary Medical Indication/Disease/Condition *

e.g. "Stress", "Diabetes", or define the target group in brackets after the condition, e.g. "Autism (Parents of children with)", "Alzheimers (Informal Caregivers of)"

difficulties in emotion regulation

Primary Outcomes measured in trial *

comma-separated list of primary outcomes reported in the trial

difficulties in emotion regulation

Secondary/other outcomes

Are there any other outcomes the intervention is expected to affect?

mental distress, well-being

Recommended "Dose" *

What do the instructions for users say on how often the app should be used?

- (selected) Approximately Daily
- Approximately Weekly
- Approximately Monthly
- Approximately Yearly
- "as needed"
- Other:

Approx. Percentage of Users (starters) still using the app as recommended after 3 months *

- (selected) unknown / not evaluated
- 0-10%
- 11-20%
- 21-30%
- 31-40%
- 41-50%
- 51-60%
- 61-70%
- 71%-80%
- 81-90%
- 91-100%
- Other:

Overall, was the app/intervention effective? *

- ○ yes: all primary outcomes were significantly better in intervention group vs control
- ○ partly: SOME primary outcomes were significantly better in intervention group vs control
- ◉ no statistically significant difference between control and intervention
- ○ potentially harmful: control was significantly better than intervention in one or more outcomes
- ○ inconclusive: more research is needed
- ○ Other:

Article Preparation Status/Stage *

At which stage in your article preparation are you currently (at the time you fill in this form)

- ○ not submitted yet - in early draft status
- ○ not submitted yet - in late draft status, just before submission
- ○ submitted to a journal but not reviewed yet
- ○ submitted to a journal and after receiving initial reviewer comments
- ◉ submitted to a journal and accepted, but not published yet
- ○ published
- ○ Other:

Journal *

If you already know where you will submit this paper (or if it is already submitted), please provide the journal name (if it is not JMIR, provide the journal name under "other")

- ○ not submitted yet / unclear where I will submit this
- ○ Journal of Medical Internet Research (JMIR)
- ◉ JMIR mHealth and UHealth
- ○ JMIR Serious Games
- ○ JMIR Mental Health
- ○ JMIR Public Health
- ○ JMIR Formative Research
- ○ Other JMIR sister journal
- ○ Other:

Is this a full powered effectiveness trial or a pilot/feasibility trial? *

- ○ Pilot/feasibility
- ◉ Fully powered

Manuscript tracking number *

If this is a JMIR submission, please provide the manuscript tracking number under "other" (The ms tracking number can be found in the submission acknowledgement email, or when you login as author in JMIR. If the paper is already published in JMIR, then the ms tracking number is the four-digit number at the end of the DOI, to be found at the bottom of each published article in JMIR)

- ○ no ms number (yet) / not (yet) submitted to / published in JMIR
- ◉ Other: JMU ms#11482

TITLE AND ABSTRACT

1a) TITLE: Identification as a randomized trial in the title

1a) Does your paper address CONSORT item 1a? *

I.e does the title contain the phrase "Randomized Controlled Trial"? (if not, explain the reason under "other")

- (•) yes
- ( ) Other:

1a-i) Identify the mode of delivery in the title

Identify the mode of delivery. Preferably use “web-based” and/or “mobile” and/or “electronic game” in the title. Avoid ambiguous terms like “online”, “virtual”, “interactive”. Use “Internet-based” only if Intervention includes non-web-based Internet components (e.g. email), use “computer-based” or “electronic” only if offline products are used. Use “virtual” only in the context of “virtual reality” (3-D worlds). Use “online” only in the context of “online support groups”. Complement or substitute product names with broader terms for the class of products (such as “mobile” or “smart phone” instead of “iphone”), especially if the application runs on different platforms.

	1	2	3	4	5	
subitem not at all important	( )	( )	( )	( )	(•)	essential

Does your paper address subitem 1a-i? *

Copy and paste relevant sections from manuscript title (include quotes in quotation marks "like this" to indicate direct quotes from your manuscript), or elaborate on this item by providing additional information not in the ms, or briefly explain why the item is not applicable/relevant for your study

"Mobile App" in title

1a-ii) Non-web-based components or important co-interventions in title

Mention non-web-based components or important co-interventions in title, if any (e.g., “with telephone support”).

	1	2	3	4	5	
subitem not at all important	(•)	( )	( )	( )	( )	essential

Does your paper address subitem 1a-ii?

Copy and paste relevant sections from manuscript title (include quotes in quotation marks "like this" to indicate direct quotes from your manuscript), or elaborate on this item by providing additional information not in the ms, or briefly explain why the item is not applicable/relevant for your study

N/A

1a-iii) Primary condition or target group in the title

Mention primary condition or target group in the title, if any (e.g., “for children with Type I Diabetes”)
Example: A Web-based and Mobile Intervention with Telephone Support for Children with Type I Diabetes: Randomized Controlled Trial

	1	2	3	4	5	
subitem not at all important	○	○	○	○	◉	essential

Does your paper address subitem 1a-iii? *

Copy and paste relevant sections from manuscript title (include quotes in quotation marks "like this" to indicate direct quotes from your manuscript), or elaborate on this item by providing additional information not in the ms, or briefly explain why the item is not applicable/relevant for your study

"Distressed Young People"

1b) ABSTRACT: Structured summary of trial design, methods, results, and conclusions

NPT extension: Description of experimental treatment, comparator, care providers, centers, and blinding status.

1b-i) Key features/functionalities/components of the intervention and comparator in the METHODS section of the ABSTRACT

Mention key features/functionalities/components of the intervention and comparator in the abstract. If possible, also mention theories and principles used for designing the site. Keep in mind the needs of systematic reviewers and indexers by including important synonyms. (Note: Only report in the abstract what the main paper is reporting. If this information is missing from the main body of text, consider adding it)

	1	2	3	4	5	
subitem not at all important	○	○	◉	○	○	essential

Does your paper address subitem 1b-i? *

Copy and paste relevant sections from the manuscript abstract (include quotes in quotation marks "like this" to indicate direct quotes from your manuscript), or elaborate on this item by providing additional information not in the ms, or briefly explain why the item is not applicable/relevant for your study

"The Music eScape mobile app teaches young people how to identify and manage emotions using music."

1b-ii) Level of human involvement in the METHODS section of the ABSTRACT

Clarify the level of human involvement in the abstract, e.g., use phrases like "fully automated" vs. "therapist/nurse/care provider/physician-assisted" (mention number and expertise of providers involved, if any). (Note: Only report in the abstract what the main paper is reporting. If this information is missing from the main body of text, consider adding it)

	1	2	3	4	5	
subitem not at all important	(●)	( )	( )	( )	( )	essential

Does your paper address subitem 1b-ii?

Copy and paste relevant sections from the manuscript abstract (include quotes in quotation marks "like this" to indicate direct quotes from your manuscript), or elaborate on this item by providing additional information not in the ms, or briefly explain why the item is not applicable/relevant for your study

No

1b-iii) Open vs. closed, web-based (self-assessment) vs. face-to-face assessments in the METHODS section of the ABSTRACT

Mention how participants were recruited (online vs. offline), e.g., from an open access website or from a clinic or a closed online user group (closed usergroup trial), and clarify if this was a purely web-based trial, or there were face-to-face components (as part of the intervention or for assessment). Clearly say if outcomes were self-assessed through questionnaires (as common in web-based trials). Note: In traditional offline trials, an open trial (open-label trial) is a type of clinical trial in which both the researchers and participants know which treatment is being administered. To avoid confusion, use "blinded" or "unblinded" to indicated the level of blinding instead of "open", as "open" in web-based trials usually refers to "open access" (i.e. participants can self-enrol). (Note: Only report in the abstract what the main paper is reporting. If this information is missing from the main body of text, consider adding it)

	1	2	3	4	5	
subitem not at all important	(●)	( )	( )	( )	( )	essential

Does your paper address subitem 1b-iii?

Copy and paste relevant sections from the manuscript abstract (include quotes in quotation marks "like this" to indicate direct quotes from your manuscript), or elaborate on this item by providing additional information not in the ms, or briefly explain why the item is not applicable/relevant for your study

No

1b-iv) RESULTS section in abstract must contain use data

Report number of participants enrolled/assessed in each group, the use/uptake of the intervention (e.g., attrition/adherence metrics, use over time, number of logins etc.), in addition to primary/secondary outcomes. (Note: Only report in the abstract what the main paper is reporting. If this information is missing from the main body of text, consider adding it)

	1	2	3	4	5	
subitem not at all important	○	○	○	●	○	essential

Does your paper address subitem 1b-iv?

Copy and paste relevant sections from the manuscript abstract (include quotes in quotation marks "like this" to indicate direct quotes from your manuscript), or elaborate on this item by providing additional information not in the ms, or briefly explain why the item is not applicable/relevant for your study

"A randomized controlled trial compared immediate versus 1-month delayed access to Music eScape in 169 young people (aged 16 to 25 years)"

1b-v) CONCLUSIONS/DISCUSSION in abstract for negative trials

Conclusions/Discussions in abstract for negative trials: Discuss the primary outcome - if the trial is negative (primary outcome not changed), and the intervention was not used, discuss whether negative results are attributable to lack of uptake and discuss reasons. (Note: Only report in the abstract what the main paper is reporting. If this information is missing from the main body of text, consider adding it)

	1	2	3	4	5	
subitem not at all important	○	○	●	○	○	essential

Does your paper address subitem 1b-v?

Copy and paste relevant sections from the manuscript abstract (include quotes in quotation marks "like this" to indicate direct quotes from your manuscript), or elaborate on this item by providing additional information not in the ms, or briefly explain why the item is not applicable/relevant for your study

"No significant differences between immediate and delayed groups on emotion regulation, distress, or well-being were found at 1 month. Both groups achieved significant improvements in 5 of the 6 emotion regulation skills, mental distress, and well-being at 2, 3, and 6 months."

INTRODUCTION

2a) In INTRODUCTION: Scientific background and explanation of rationale

2a-i) Problem and the type of system/solution

Describe the problem and the type of system/solution that is object of the study: intended as stand-alone intervention vs. incorporated in broader health care program? Intended for a particular patient population? Goals of the intervention, e.g., being more cost-effective to other interventions, replace or complement other solutions? (Note: Details about the intervention are provided in "Methods" under 5)

	1	2	3	4	5	
subitem not at all important	○	○	○	○	●	essential

Does your paper address subitem 2a-i? *

Copy and paste relevant sections from the manuscript (include quotes in quotation marks "like this" to indicate direct quotes from your manuscript), or elaborate on this item by providing additional information not in the ms, or briefly explain why the item is not applicable/relevant for your study

"Mental health and substance use disorders are at their peak and are the leading causes of disability and death worldwide in young people (aged 15-25 years) [1]. Anxiety, depression, and substance use disorders are the most common in this age group. Deficits in emotion regulation or the ability to identify, evaluate, express, and modify emotions are important risk and maintaining factors for these disorders [2-5]. Emotion regulation requires the development and integration of several emotion skills such as an awareness of one's emotional states, directing one's attention away from the cause of a negative emotion,; cognitively reappraising the cause of the negative emotion,; and accepting, discharging, or suppressing a negative emotional state [6,7]. Young people commonly experience intense emotional states during puberty and the transition from childhood to adulthood [8]. At the same time, their capacity to fully regulate emotional states is still developing [9,10]. Effective emotion regulation skills may help decrease the intensity and duration of dysphoric states, whereas ineffective emotion regulation may increase them [3].

Only a third of young people with mental disorders seek help [11] and many fail to engage in or complete psychological treatment. Around 60% of young people with anxiety disorders respond to cognitive behavioral therapy, with response rates for unipolar major depression ranging from 48% to 81% over 36 weeks [12-15]. Nevertheless, around 40% of young people with anxiety and 20% to 25% with depressive disorders do not respond to treatment [13,15-17], and 47% of responders to depression treatment relapse within 5 years [18].

Novel interventions targeting young people's emotion regulation skills could reduce the risk of anxiety or depression disorders and improve help seeking, treatment engagement, and outcomes among those with these disorders. Music listening is one of the favorite leisure activities of young people [19] and one of their most commonly used emotion regulation strategies [20-27], being recently ranked the top stress management strategy in young Australians [28].

Music is commonly used by young people and others to induce, enhance, maintain, or manage moods [29-31]. The impact of music on mood varies according to the goal of the user [32], their pre-existing mood [33], and the type of emotion regulation strategy being used [34]. For example, a correlational study found substantial proportions of adolescents reported improvements in mood when listening to music; these effects were most pronounced when they were happy or bored, rather than angry or sad [33]. Positive effects of music on negative moods appeared constrained by some adolescents preferring angry rather than happy music when in a negative mood [33]. A Web-based experiment found listening to self-selected sad music increased depressive moods and listening to happy music reduced them [34]. However, a partial replication of that

study found both participant- and experimenter-selected sad music reduced a depressive mood if a negative mood was induced (via a video clip) before music listening [35]. Finally, music use among people who use cognitive reappraisal as an emotion regulation strategy has been found to enhance well-being, whereas the use of expressive suppression reduced well-being [36]. In summary, music appears to have potential to be used as an effective emotion regulation skill for improving mood and well-being, although the relationship is complex and controlled studies of adequate power are needed [37]."

2a-ii) Scientific background, rationale: What is known about the (type of) system

Scientific background, rationale: What is known about the (type of) system that is the object of the study (be sure to discuss the use of similar systems for other conditions/diagnoses, if appropiate), motivation for the study, i.e. what are the reasons for and what is the context for this specific study, from which stakeholder viewpoint is the study performed, potential impact of findings [2]. Briefly justify the choice of the comparator.

	1	2	3	4	5	
subitem not at all important	○	○	○	○	◉	essential

Does your paper address subitem 2a-ii? *

Copy and paste relevant sections from the manuscript (include quotes in quotation marks "like this" to indicate direct quotes from your manuscript), or elaborate on this item by providing additional information not in the ms, or briefly explain why the item is not applicable/relevant for your study

"The relationship between music and emotions has been harnessed in programs aimed at teaching emotion regulation skills to individuals with mental health problems in clinical and community settings, including eating disorders [38], anxiety disorders [39], substance misuse [40], and schizophrenia [41]. One example is the Tuned In group program, which uses hypothetical scenarios and participant-selected music to evoke emotions in sessions to increase emotional awareness and emotion regulation skills. This program demonstrated significant improvements in emotional awareness and regulation post treatment among 41 at-risk adolescents attending an education re-engagement program and 216 adolescents attending an independent mainstream secondary school [42]. A 4-session version of the program among dysphoric first-year university students (n = 51; aged 18 - 25 years) found greater emotional awareness and regulation post treatment compared with a 4-week waitlist control group [43]. These findings provide preliminary evidence that music programs such as Tuned In result in positive emotion regulation outcomes.

Media has the potential to enhance the emotion regulation skills and mood of young people in their everyday lives. For instance, a survey study of 229 people found that mood-specific media use might be captured by 3 factors: turning to media in a positive mood, in a negative mood, or in a bored mood [10]. Various forms of difficulty regulating emotion (e.g., feeling out of control when upset) predicted media use in negative or bored moods only. More specific analyses show that music use in negative moods is predicted by both positive indices (e.g., reflection tendencies) and negative indices of emotion regulation (e.g., rumination tendencies), whereas television use in negative moods is only predicted by negative indices of emotion regulation [10]. A systemic review of 23 studies on the use of video games for emotion regulation reported that frequent (but not excessive) video game play, including serious games, may enhance emotion regulation, but commercial gaming offered more opportunities for emotion regulation improvement than limited-time (bespoke) games [44].

Music also has the potential to enhance the emotion regulation skills and mood of young people in their everyday lives. Mobile phones that contain digital music players, personal music libraries, and access to digital radio provide a platform for achieving this. Targeted music apps, therefore, provide an anonymous and highly accessible way of providing young people with the skills to identify, express, and manage emotions in their natural environment [19,45,46]. A recent meta-analysis of 21 studies of electronic health (eHealth) interventions for youth concluded that such apps could result in population-level benefits, even with small effects (d = 0.13; 95% CI 0.02 - 0.25) [47].

A growing number of mobile phone apps targeting emotions through music are

becoming available. Several approaches are used, including streaming mood-related playlists, providing mood-tagging options for users' own libraries, and playing sounds and soundscapes to promote relaxation [31]. However, it is unclear how the music mood ratings contained in these are derived, and the majority are not specifically designed to help young people regulate emotions."

2b) In INTRODUCTION: Specific objectives or hypotheses

Does your paper address CONSORT subitem 2b? *

Copy and paste relevant sections from the manuscript (include quotes in quotation marks "like this" to indicate direct quotes from your manuscript), or elaborate on this item by providing additional information not in the ms, or briefly explain why the item is not applicable/relevant for your study

"This study aimed to evaluate a new app called Music eScape, developed to assist young people with identifying, expressing, and managing emotions using music from their own music library. This study reports the 1-month efficacy and 2-, 3-, and 6-month outcomes of the Music eScape app in a sample of young people with at least mild mental distress. Potential moderators of app outcomes, including the amount of music use and healthy or unhealthy music use, were examined. In addition, user ratings of the app's quality were obtained after a month of its use."

METHODS

3a) Description of trial design (such as parallel, factorial) including allocation ratio

Does your paper address CONSORT subitem 3a? *

Copy and paste relevant sections from the manuscript (include quotes in quotation marks "like this" to indicate direct quotes from your manuscript), or elaborate on this item by providing additional information not in the ms, or briefly explain why the item is not applicable/relevant for your study

"immediate- or delayed-access groups, with stratification by age group (aged 16-20 years and 21-25 years) and gender"

3b) Important changes to methods after trial commencement (such as eligibility criteria), with reasons

Does your paper address CONSORT subitem 3b? *

Copy and paste relevant sections from the manuscript (include quotes in quotation marks "like this" to indicate direct quotes from your manuscript), or elaborate on this item by providing additional information not in the ms, or briefly explain why the item is not applicable/relevant for your study

Not relevant - no changes were implemented to methods after study commencement.

3b-i) Bug fixes, Downtimes, Content Changes

Bug fixes, Downtimes, Content Changes: ehealth systems are often dynamic systems. A description of changes to methods therefore also includes important changes made on the intervention or comparator during the trial (e.g., major bug fixes or changes in the functionality or content) (5-iii) and other "unexpected events" that may have influenced study design such as staff changes, system failures/downtimes, etc. [2].

	1	2	3	4	5	
subitem not at all important	○	○	○	○	◉	essential

Does your paper address subitem 3b-i?

Copy and paste relevant sections from the manuscript (include quotes in quotation marks "like this" to indicate direct quotes from your manuscript), or elaborate on this item by providing additional information not in the ms, or briefly explain why the item is not applicable/relevant for your study

No changes to the intervention were done during the trial. Individuals who required assistance with app download were able to contact the research team for support.

4a) Eligibility criteria for participants

Does your paper address CONSORT subitem 4a? *

Copy and paste relevant sections from the manuscript (include quotes in quotation marks "like this" to indicate direct quotes from your manuscript), or elaborate on this item by providing additional information not in the ms, or briefly explain why the item is not applicable/relevant for your study

"Participants were Australian residents aged 16 to 25 years, who reported at least mild distress in the past month on the Kessler 10 Psychological Distress scale (K10 > 17) and had an iPhone. Recruitment was via student emails and posters in 2 large universities, and snowballing techniques. The advertisements invited young people (aged 16 - 25 years) who owned an iPhone and felt stressed to participate in a study testing a new mood management app. They did not include any mention about music in an attempt to avoid recruiting a selective sample of participants with a high affinity to music. The purpose of the study was also concealed during the consent process, such that participants were not aware of the fact that the mood management app used music until they received access to it."

4a-i) Computer / Internet literacy

Computer / Internet literacy is often an implicit "de facto" eligibility criterion - this should be explicitly clarified.

	1	2	3	4	5	
subitem not at all important	○	○	○	○	◉	essential

Does your paper address subitem 4a-i?

Copy and paste relevant sections from the manuscript (include quotes in quotation marks "like this" to indicate direct quotes from your manuscript), or elaborate on this item by providing additional information not in the ms, or briefly explain why the item is not applicable/relevant for your study

"had an iPhone" was considered as sufficient literacy level requirement.

4a-ii) Open vs. closed, web-based vs. face-to-face assessments:

Open vs. closed, web-based vs. face-to-face assessments: Mention how participants were recruited (online vs. offline), e.g., from an open access website or from a clinic, and clarify if this was a purely web-based trial, or there were face-to-face components (as part of the intervention or for assessment), i.e., to what degree got the study team to know the participant. In online-only trials, clarify if participants were quasi-anonymous and whether having multiple identities was possible or whether technical or logistical measures (e.g., cookies, email confirmation, phone calls) were used to detect/prevent these.

	1	2	3	4	5	
subitem not at all important	○	○	○	○	◉	essential

Does your paper address subitem 4a-ii? *

Copy and paste relevant sections from the manuscript (include quotes in quotation marks "like this" to indicate direct quotes from your manuscript), or elaborate on this item by providing additional information not in the ms, or briefly explain why the item is not applicable/relevant for your study

"Recruitment was via student emails and posters in 2 large universities, and snowballing techniques."
"Those assigned to the immediate-access group were emailed a link to the app. This required users to first download the TestFlight app, a beta app distribution platform, which enabled download of the Music eScape app before its release in the iPhone store. Short message service (SMS) text message reminders to access the app were sent at 7-day intervals in the first month.
To minimize attrition, the delayed-access group received 2 SMS text messages during the 1 month wait for access to the app. All baseline and follow-up surveys were completed online. Participants were automatically sent email links to each survey 3 days before, on the day of, and at 3 and 7 days after a follow up was due. Reminder SMS text messages were sent to those who had not completed a follow-up, 8 and 10 days after they were due. Participants were reimbursed Aus $20 for completing each survey."

4a-iii) Information giving during recruitment

Information given during recruitment. Specify how participants were briefed for recruitment and in the informed consent procedures (e.g., publish the informed consent documentation as appendix, see also item X26), as this information may have an effect on user self-selection, user expectation and may also bias results.

	1	2	3	4	5	
subitem not at all important	○	○	○	○	◉	essential

Does your paper address subitem 4a-iii?

Copy and paste relevant sections from the manuscript (include quotes in quotation marks "like this" to indicate direct quotes from your manuscript), or elaborate on this item by providing additional information not in the ms, or briefly explain why the item is not applicable/relevant for your study

"The advertisements invited young people (aged 16 - 25 years) who owned an iPhone and felt stressed to participate in a study testing a new mood management app. They did not include any mention about music in an attempt to avoid recruiting a selective sample of participants with a high affinity to music. The purpose of the study was also concealed during the consent process, such that participants were not aware of the fact that the mood management app used music until they received access to it."

4b) Settings and locations where the data were collected

Does your paper address CONSORT subitem 4b? *

Copy and paste relevant sections from the manuscript (include quotes in quotation marks "like this" to indicate direct quotes from your manuscript), or elaborate on this item by providing additional information not in the ms, or briefly explain why the item is not applicable/relevant for your study

"All baseline and follow-up surveys were completed online. Participants were automatically sent email links to each survey 3 days before, on the day of, and at 3 and 7 days after a follow up was due. Reminder SMS text messages were sent to those who had not completed a follow-up, 8 and 10 days after they were due. Participants were reimbursed Aus $20 for completing each survey."

4b-i) Report if outcomes were (self-)assessed through online questionnaires

Clearly report if outcomes were (self-)assessed through online questionnaires (as common in web-based trials) or otherwise.

	1	2	3	4	5	
subitem not at all important	○	○	○	○	◉	essential

Does your paper address subitem 4b-i? *

Copy and paste relevant sections from the manuscript (include quotes in quotation marks "like this" to indicate direct quotes from your manuscript), or elaborate on this item by providing additional information not in the ms, or briefly explain why the item is not applicable/relevant for your study

As above

4b-ii) Report how institutional affiliations are displayed

Report how institutional affiliations are displayed to potential participants [on ehealth media], as affiliations with prestigious hospitals or universities may affect volunteer rates, use, and reactions with regards to an intervention.(Not a required item – describe only if this may bias results)

	1	2	3	4	5	
subitem not at all important	◉	○	○	○	○	essential

Does your paper address subitem 4b-ii?

Copy and paste relevant sections from the manuscript (include quotes in quotation marks "like this" to indicate direct quotes from your manuscript), or elaborate on this item by providing additional information not in the ms, or briefly explain why the item is not applicable/relevant for your study

N/A

5) The interventions for each group with sufficient details to allow replication, including how and when they were actually administered

5-i) Mention names, credential, affiliations of the developers, sponsors, and owners

Mention names, credential, affiliations of the developers, sponsors, and owners [6] (if authors/evaluators are owners or developer of the software, this needs to be declared in a “Conflict of interest” section or mentioned elsewhere in the manuscript).

	1	2	3	4	5	
subitem not at all important	○	◉	○	○	○	essential

Does your paper address subitem 5-i?

Copy and paste relevant sections from the manuscript (include quotes in quotation marks "like this" to indicate direct quotes from your manuscript), or elaborate on this item by providing additional information not in the ms, or briefly explain why the item is not applicable/relevant for your study

"The Young and Well Cooperative Research Centre (Young and Well CRC; 2011-2016) funded this project. The Young and Well CRC was an Australian-based international research center established under the Australian Government’s Cooperative Research Centres Program. These funders had no role in the study design; collection, analysis, or interpretation of data; writing the manuscript; and the decision to submit the manuscript for publication."

5-ii) Describe the history/development process

Describe the history/development process of the application and previous formative evaluations (e.g., focus groups, usability testing), as these will have an impact on adoption/use rates and help with interpreting results.

	1	2	3	4	5	
subitem not at all important	○	○	○	○	◉	essential

Does your paper address subitem 5-ii?

Copy and paste relevant sections from the manuscript (include quotes in quotation marks "like this" to indicate direct quotes from your manuscript), or elaborate on this item by providing additional information not in the ms, or briefly explain why the item is not applicable/relevant for your study

"The Music eScape app was co-designed by young people and a multidisciplinary research team using a series of participatory design workshops [48]. App design was informed by the dynamic information-motivation-behavioural skills health behaviour model [49,50], and agile development processes were used. "

5-iii) Revisions and updating

Revisions and updating. Clearly mention the date and/or version number of the application/intervention (and comparator, if applicable) evaluated, or describe whether the intervention underwent major changes during the evaluation process, or whether the development and/or content was "frozen" during the trial. Describe dynamic components such as news feeds or changing content which may have an impact on the replicability of the intervention (for unexpected events see item 3b).

	1	2	3	4	5	
subitem not at all important	◉	○	○	○	○	essential

Does your paper address subitem 5-iii?

Copy and paste relevant sections from the manuscript (include quotes in quotation marks "like this" to indicate direct quotes from your manuscript), or elaborate on this item by providing additional information not in the ms, or briefly explain why the item is not applicable/relevant for your study

No changes to the intervention were done during the trial.

5-iv) Quality assurance methods

Provide information on quality assurance methods to ensure accuracy and quality of information provided [1], if applicable.

	1	2	3	4	5	
subitem not at all important	◉	○	○	○	○	essential

Does your paper address subitem 5-iv?

Copy and paste relevant sections from the manuscript (include quotes in quotation marks "like this" to indicate direct quotes from your manuscript), or elaborate on this item by providing additional information not in the ms, or briefly explain why the item is not applicable/relevant for your study

Continuous testing of the intervention and process was conducted before commencing the trial. However, this process is not reported in the manuscript.

5-v) Ensure replicability by publishing the source code, and/or providing screenshots/screen-capture video, and/or providing flowcharts of the algorithms used

Ensure replicability by publishing the source code, and/or providing screenshots/screen-capture video, and/or providing flowcharts of the algorithms used. Replicability (i.e., other researchers should in principle be able to replicate the study) is a hallmark of scientific reporting.

	1	2	3	4	5	
subitem not at all important	◉	○	○	○	○	essential

Does your paper address subitem 5-v?

Copy and paste relevant sections from the manuscript (include quotes in quotation marks "like this" to indicate direct quotes from your manuscript), or elaborate on this item by providing additional information not in the ms, or briefly explain why the item is not applicable/relevant for your study

Source code for the app used in the intervention is subject IP regulations of the participating developer institutions.

5-vi) Digital preservation

Digital preservation: Provide the URL of the application, but as the intervention is likely to change or disappear over the course of the years; also make sure the intervention is archived (Internet Archive, webcitation.org, and/or publishing the source code or screenshots/videos alongside the article). As pages behind login screens cannot be archived, consider creating demo pages which are accessible without login.

	1	2	3	4	5	
subitem not at all important	○	○	◉	○	○	essential

Does your paper address subitem 5-vi?

Copy and paste relevant sections from the manuscript (include quotes in quotation marks "like this" to indicate direct quotes from your manuscript), or elaborate on this item by providing additional information not in the ms, or briefly explain why the item is not applicable/relevant for your study

While we have provided relevant screenshots, the application web page on the Apple App Store is no longer available.

5-vii) Access

Access: Describe how participants accessed the application, in what setting/context, if they had to pay (or were paid) or not, whether they had to be a member of specific group. If known, describe how participants obtained “access to the platform and Internet” [1]. To ensure access for editors/reviewers/readers, consider to provide a “backdoor” login account or demo mode for reviewers/readers to explore the application (also important for archiving purposes, see vi).

	1	2	3	4	5	
subitem not at all important	○	○	○	○	◉	essential

Does your paper address subitem 5-vii? *

Copy and paste relevant sections from the manuscript (include quotes in quotation marks "like this" to indicate direct quotes from your manuscript), or elaborate on this item by providing additional information not in the ms, or briefly explain why the item is not applicable/relevant for your study

"This required users to first download the TestFlight app, a beta app distribution platform, which enabled download of the Music eScape app before its release in the Apple App Store."

5-viii) Mode of delivery, features/functionalities/components of the intervention and comparator, and the theoretical framework

Describe mode of delivery, features/functionalities/components of the intervention and comparator, and the theoretical framework [6] used to design them (instructional strategy [1], behaviour change techniques, persuasive features, etc., see e.g., [7, 8] for terminology). This includes an in-depth description of the content (including where it is coming from and who developed it) [1],” whether [and how] it is tailored to individual circumstances and allows users to track their progress and receive feedback” [6]. This also includes a description of communication delivery channels and – if computer-mediated communication is a component – whether communication was synchronous or asynchronous [6]. It also includes information on presentation strategies [1], including page design principles, average amount of text on pages, presence of hyperlinks to other resources, etc. [1].

	1	2	3	4	5	
subitem not at all important	○	○	○	○	◉	essential

Does your paper address subitem 5-viii? *

Copy and paste relevant sections from the manuscript (include quotes in quotation marks "like this" to indicate direct quotes from your manuscript), or elaborate on this item by providing additional information not in the ms, or briefly explain why the item is not applicable/relevant for your study

"The Music eScape app analyzes each song in the users' music library according to its level of valence (pleasant to unpleasant) and arousal (very low to very high) using The Echo Nest music data program [70]. The songs are then located in a two-dimensional space consistent with Russell's circumplex model of emotion [51], labeled around the borders with 8 emotions (see Figure 1, left screenshot): aggressive, excited, happy, chilled, peaceful, bored, depressed, and stressed. Once the music scanning is complete, the user is presented with a mood map of their music (the eScape) to help them identify the prevalent moods of their library (see Figure 1, middle screenshot). Before creating a playlist, the app prompts users to reflect on their current and desired mood and then encourages them to plot a mood journey the playlist will support them to create (see Figure 1, right screenshot). This journey comprises a unique trajectory using their own music (eg, starting in the bored segment and ending in the happy one; see Figure 1, right screenshot). Users can save and label their musical mood journeys (eg, Chill out) and can also specify the duration of their playlist, from 15 min to 60 min. They can also select preset mood journeys. After completing their mood journey, users are asked to reflect on their current mood and rate the effectiveness of the playlist they just experienced."

5-ix) Describe use parameters

Describe use parameters (e.g., intended "doses" and optimal timing for use). Clarify what instructions or recommendations were given to the user, e.g., regarding timing, frequency, heaviness of use, if any, or was the intervention used ad libitum.

	1	2	3	4	5	
subitem not at all important	(●)	○	○	○	○	essential

Does your paper address subitem 5-ix?

Copy and paste relevant sections from the manuscript (include quotes in quotation marks "like this" to indicate direct quotes from your manuscript), or elaborate on this item by providing additional information not in the ms, or briefly explain why the item is not applicable/relevant for your study

As we aimed to keep measure ad libitum use of the app, no specific parameters were recommended to participants. The only aspect that may have affected use is described in the paper as follows: "Short message service (SMS) text message reminders to access the app were sent at 7-day intervals in the first month."

5-x) Clarify the level of human involvement

Clarify the level of human involvement (care providers or health professionals, also technical assistance) in the e-intervention or as co-intervention (detail number and expertise of professionals involved, if any, as well as “type of assistance offered, the timing and frequency of the support, how it is initiated, and the medium by which the assistance is delivered”. It may be necessary to distinguish between the level of human involvement required for the trial, and the level of human involvement required for a routine application outside of a RCT setting (discuss under item 21 – generalizability).

	1	2	3	4	5	
subitem not at all important	○	○	○	○	◉	essential

Does your paper address subitem 5-x?

Copy and paste relevant sections from the manuscript (include quotes in quotation marks "like this" to indicate direct quotes from your manuscript), or elaborate on this item by providing additional information not in the ms, or briefly explain why the item is not applicable/relevant for your study

No health professionals have contacted the study participants. Data collection and app access were automated via an automated, customized, university-developed RCT-management program similar to Redcap. The following text is available in the manuscript: "All baseline and follow-up surveys were completed online. Participants were automatically sent email links to each survey 3 days before, on the day of, and at 3 and 7 days after a follow up was due. Reminder SMS text messages were sent to those who had not completed a follow-up, 8 and 10 days after they were due. ".
Participants were contacted only if they had sent an inquiry to the research team regarding the process of app download, or if they had reported experiencing difficulties with app use.

5-xi) Report any prompts/reminders used

Report any prompts/reminders used: Clarify if there were prompts (letters, emails, phone calls, SMS) to use the application, what triggered them, frequency etc. It may be necessary to distinguish between the level of prompts/reminders required for the trial, and the level of prompts/reminders for a routine application outside of a RCT setting (discuss under item 21 – generalizability).

	1	2	3	4	5	
subitem not at all important	○	○	○	○	◉	essential

Does your paper address subitem 5-xi? *

Copy and paste relevant sections from the manuscript (include quotes in quotation marks "like this" to indicate direct quotes from your manuscript), or elaborate on this item by providing additional information not in the ms, or briefly explain why the item is not applicable/relevant for your study

As above

5-xii) Describe any co-interventions (incl. training/support)

Describe any co-interventions (incl. training/support): Clearly state any interventions that are provided in addition to the targeted eHealth intervention, as ehealth intervention may not be designed as stand-alone intervention. This includes training sessions and support [1]. It may be necessary to distinguish between the level of training required for the trial, and the level of training for a routine application outside of a RCT setting (discuss under item 21 – generalizability.

	1	2	3	4	5	
subitem not at all important	◉	○	○	○	○	essential

Does your paper address subitem 5-xii? *

Copy and paste relevant sections from the manuscript (include quotes in quotation marks "like this" to indicate direct quotes from your manuscript), or elaborate on this item by providing additional information not in the ms, or briefly explain why the item is not applicable/relevant for your study

N/A

6a) Completely defined pre-specified primary and secondary outcome measures, including how and when they were assessed

Does your paper address CONSORT subitem 6a? *

Copy and paste relevant sections from the manuscript (include quotes in quotation marks "like this" to indicate direct quotes from your manuscript), or elaborate on this item by providing additional information not in the ms, or briefly explain why the item is not applicable/relevant for your study

"primary outcome variable of difficulties in emotion regulation and secondary outcomes of mental distress and well-being. For all outcomes, time and group main effects, and time by group interaction from baseline to 1 month were conducted, followed by analyses examining the impact of the app over time from baseline to the 2-, 3-, and 6-month follow-ups."

6a-i) Online questionnaires: describe if they were validated for online use and apply CHERRIES items to describe how the questionnaires were designed/deployed

If outcomes were obtained through online questionnaires, describe if they were validated for online use and apply CHERRIES items to describe how the questionnaires were designed/deployed [9].

	1	2	3	4	5	
subitem not at all important	○	○	○	○	◉	essential

Does your paper address subitem 6a-i?

Copy and paste relevant sections from manuscript text

"Emotion Regulation

The 18-item short-form of the Difficulties in Emotion Regulation Scale (DERS-SF) [52] was used to assesses emotion dysregulation on a scale from 1 (almost never) to 5 (almost always). The DERS-SF has excellent reliability and validity and a similar factor structure to the original 36-item scale in adolescents and adults [53]. There are 6 subscales: lack of emotional awareness (alpha=.80), lack of emotional clarity (alpha=.83), difficulties engaging in goal-directed behaviors (alpha=.88), impulse control difficulties (alpha=.91), nonacceptance of emotional responses (alpha=.85), and limited access to emotion regulation strategies (alpha=.85). Participants were also asked to rate their perceived level of success with using music as an emotion regulation strategy on a Likert scale (1=not at all successful to 9=extremely successful; item derived from Thayer et al's study) [54].

Mental Distress and Well-Being

The K10 scale [55] assessed the frequency of psychological distress in the past month, using items rated from 1 (none of the time) to 5 (all of the time). The K10 is a widely used screening tool developed using item response theory to determine the probable presence or absence of a diagnosable anxiety or depressive disorder. Normative data indicate that a cut-off of ≥17 is indicative of at least mild mental distress and is at the seventy-fifth percentile among Australian youth (aged 16-24 years) [56]. Internal consistency was high in this study sample (alpha=.87).

Mental well-being was measured with the Mental Health Continuum-Short Form (MHC-SF) [57,58]. This 14-item scale measures how frequently respondents experienced emotional, psychological, and social well-being in the past month on a 6-point response scale (1=never to 6=every day). The MHC-SF has high levels of reliability and discriminant, convergent, and cross-cultural validity. Internal consistency was .94.

Music Measures

A total of 10 items designed specifically for this study explored the level of music education and involvement of participants. Example items include the following: "Do you currently play a musical instrument and/or sing in a group or choir?" (yes or no) and "Do you attend concerts or live music on a regular basis (ie, at least once a month)?" (yes or no).

The Healthy-Unhealthy Music Scale [59] assesses healthy (5 items) and unhealthy (8 items) uses of music, with items rated from 1 (never) to 5 (always). Healthy and unhealthy music use refers to protective (eg, "Music gives me the energy to get going") versus risky (eg, "When I listen to music I get stuck in bad memories") forms of music engagement [59]. The healthy subscale has demonstrated concurrent validity with well-being, happiness, and school satisfaction, and its unhealthy subscale is associated with depression

satisfaction, and its unhealthy subscale is associated with depression, rumination, and stress. Internal consistency in this sample was healthy: alpha=.76 and unhealthy: alpha=.85. A median split was calculated for each of these variables to identify participants scoring either low or high on healthy and low or high on unhealthy music use."

"App engagement was defined as the total number of playlists created per participant. App quality was assessed by the Mobile App Rating Scale-User version (uMARS) [60]. This 20-item scale assesses perceived objective app quality on 4 subscales (engagement, functionality, aesthetics, and information) rated on a 5-point scale (1=very poor and 5=excellent). Mean subscale scores and a mean objective quality score were derived. Subjective app quality was assessed using 4 questions: “Would you recommend the app?” (1, not to anyone; to 5, everyone); “How many times would you use it?” (1, 0 times; to 5, >50 times); “Would you pay for this app?” (1, no; 2, maybe; and 3, yes); and overall star-rating (1 to 5 stars)."

6a-ii) Describe whether and how “use” (including intensity of use/dosage) was defined/measured/monitored

Describe whether and how “use” (including intensity of use/dosage) was defined/measured/monitored (logins, logfile analysis, etc.). Use/adoption metrics are important process outcomes that should be reported in any ehealth trial.

	1	2	3	4	5	
subitem not at all important	○	○	○	○	◉	essential

Does your paper address subitem 6a-ii?

Copy and paste relevant sections from manuscript text

"App Use and Quality
Backend data on the date, time, frequency, and length of app use were collected."

6a-iii) Describe whether, how, and when qualitative feedback from participants was obtained

Describe whether, how, and when qualitative feedback from participants was obtained (e.g., through emails, feedback forms, interviews, focus groups).

	1	2	3	4	5	
subitem not at all important	○	○	○	○	◉	essential

Does your paper address subitem 6a-iii?

Copy and paste relevant sections from manuscript text

Qualitative feedback was permitted at the end of the uMARS scale online via textbox comments. Relevant information constituted reports of difficulties with app use, which were also reflected in the backend data usage capture. Those are reported separately in the manuscript.

6b) Any changes to trial outcomes after the trial commenced, with reasons

Does your paper address CONSORT subitem 6b? *

Copy and paste relevant sections from the manuscript (include quotes in quotation marks "like this" to indicate direct quotes from your manuscript), or elaborate on this item by providing additional information not in the ms, or briefly explain why the item is not applicable/relevant for your study

N/A

7a) How sample size was determined

NPT: When applicable, details of whether and how the clustering by care provides or centers was addressed

7a-i) Describe whether and how expected attrition was taken into account when calculating the sample size

Describe whether and how expected attrition was taken into account when calculating the sample size.

	1	2	3	4	5	
subitem not at all important	●	○	○	○	○	essential

Does your paper address subitem 7a-i?

Copy and paste relevant sections from manuscript title (include quotes in quotation marks "like this" to indicate direct quotes from your manuscript), or elaborate on this item by providing additional information not in the ms, or briefly explain why the item is not applicable/relevant for your study

Sample size was calculated using GPower. Attrition was set to reach 20% over the life of the trial. Yet, lower attrition rates were recorded and are reported in the manuscript: "Follow-up rates were high (93.5% at 1 month, 87.6% at 2 months, 88.2% at 3 months, and 84.0% at 6 months)"

7b) When applicable, explanation of any interim analyses and stopping guidelines

Does your paper address CONSORT subitem 7b? *

Copy and paste relevant sections from the manuscript (include quotes in quotation marks "like this" to indicate direct quotes from your manuscript), or elaborate on this item by providing additional information not in the ms, or briefly explain why the item is not applicable/relevant for your study

N/A

8a) Method used to generate the random allocation sequence

NPT: When applicable, how care providers were allocated to each trial group

Does your paper address CONSORT subitem 8a? *

Copy and paste relevant sections from the manuscript (include quotes in quotation marks "like this" to indicate direct quotes from your manuscript), or elaborate on this item by providing additional information not in the ms, or briefly explain why the item is not applicable/relevant for your study

"Those meeting study inclusion criteria were automatically identified and randomized via a computerized trial management system to the immediate- or delayed-access groups"

8b) Type of randomisation; details of any restriction (such as blocking and block size)

Does your paper address CONSORT subitem 8b? *

Copy and paste relevant sections from the manuscript (include quotes in quotation marks "like this" to indicate direct quotes from your manuscript), or elaborate on this item by providing additional information not in the ms, or briefly explain why the item is not applicable/relevant for your study

"with stratification by age group (aged 16-20 years and 21-25 years) and

9) Mechanism used to implement the random allocation sequence (such as sequentially numbered containers), describing any steps taken to conceal the sequence until interventions were assigned

Does your paper address CONSORT subitem 9? *

Copy and paste relevant sections from the manuscript (include quotes in quotation marks "like this" to indicate direct quotes from your manuscript), or elaborate on this item by providing additional information not in the ms, or briefly explain why the item is not applicable/relevant for your study

"randomized via a computerized trial management system"

10) Who generated the random allocation sequence, who enrolled participants, and who assigned participants to interventions

Does your paper address CONSORT subitem 10? *

Copy and paste relevant sections from the manuscript (include quotes in quotation marks "like this" to indicate direct quotes from your manuscript), or elaborate on this item by providing additional information not in the ms, or briefly explain why the item is not applicable/relevant for your study

"randomized via a computerized trial management system"

11a) If done, who was blinded after assignment to interventions (for example, participants, care providers, those assessing outcomes) and how

NPT: Whether or not administering co-interventions were blinded to group assignment

11a-i) Specify who was blinded, and who wasn't

Specify who was blinded, and who wasn't. Usually, in web-based trials it is not possible to blind the participants [1, 3] (this should be clearly acknowledged), but it may be possible to blind outcome assessors, those doing data analysis or those administering co-interventions (if any).

	1	2	3	4	5	
subitem not at all important	○	○	○	○	◉	essential

Does your paper address subitem 11a-i? *

Copy and paste relevant sections from the manuscript (include quotes in quotation marks "like this" to indicate direct quotes from your manuscript), or elaborate on this item by providing additional information not in the ms, or briefly explain why the item is not applicable/relevant for your study

Due to the full automation of data-collection via the trial-managing software, and as appropriate tech support was made available to participants, no blinding was implemented. Yet, all participants received the intervention either immediately, or after a one-month delay. Thus, all participants had equal chance of receiving tech support.

11a-ii) Discuss e.g., whether participants knew which intervention was the "intervention of interest" and which one was the "comparator"

Informed consent procedures (4a-ii) can create biases and certain expectations - discuss e.g., whether participants knew which intervention was the "intervention of interest" and which one was the "comparator".

	1	2	3	4	5	
subitem not at all important	○	○	◉	○	○	essential

Does your paper address subitem 11a-ii?

Copy and paste relevant sections from the manuscript (include quotes in quotation marks "like this" to indicate direct quotes from your manuscript), or elaborate on this item by providing additional information not in the ms, or briefly explain why the item is not applicable/relevant for your study

Both groups accessed the intervention.

11b) If relevant, description of the similarity of interventions

(this item is usually not relevant for ehealth trials as it refers to similarity of a placebo or sham intervention to a active medication/intervention)

Does your paper address CONSORT subitem 11b? *

Copy and paste relevant sections from the manuscript (include quotes in quotation marks "like this" to indicate direct quotes from your manuscript), or elaborate on this item by providing additional information not in the ms, or briefly explain why the item is not applicable/relevant for your study

Identical intervention, delayed access.

12a) Statistical methods used to compare groups for primary and secondary outcomes

NPT: When applicable, details of whether and how the clustering by care providers or centers was addressed

Does your paper address CONSORT subitem 12a? *

Copy and paste relevant sections from the manuscript (include quotes in quotation marks "like this" to indicate direct quotes from your manuscript), or elaborate on this item by providing additional information not in the ms, or briefly explain why the item is not applicable/relevant for your study

"The immediate- and delayed-access groups were compared on baseline demographic, mental distress and well-being, emotion regulation, and music variables using logistic regressions, with treatment group allocation as the outcome variable. Data screening indicated all outcomes (ie, emotion regulation, mental distress, and well-being) had acceptable skew and kurtosis. Linear mixed models in SPSS version 25 (IBM Corp, Armonk, NY, USA) were used to conduct intent-to-treat analyses, without prediction of missing data, on the primary outcome variable of difficulties in emotion regulation and secondary outcomes of mental distress and well-being. For all outcomes, time and group main effects, and time by group interaction from baseline to 1 month were conducted, followed by analyses examining the impact of the app over time from baseline to the 2-, 3-, and 6-month follow-ups. Gender, baseline duration of music use (hours per week of music listening, with median split into high vs low), and use of music (healthy or unhealthy) were included as control variables and potential moderators of outcomes because of the potential impact of these variables on mood, music, and app use [59]. Two analyses entering app access (yes or no) and app use as additional control variables were also conducted to determine if this varied results. An autoregressive covariance structure (Toeplitz) was specified to account for correlated outcome variables assessed at close time points. Significant effects were probed using pairwise comparisons, and Cohen d effect sizes were calculated using SDs pooled across groups and times."

12a-i) Imputation techniques to deal with attrition / missing values

Imputation techniques to deal with attrition / missing values: Not all participants will use the intervention/comparator as intended and attrition is typically high in ehealth trials. Specify how participants who did not use the application or dropped out from the trial were treated in the statistical analysis (a complete case analysis is strongly discouraged, and simple imputation techniques such as LOCF may also be problematic [4]).

	1	2	3	4	5	
subitem not at all important	○	●	○	○	○	essential

Does your paper address subitem 12a-i? *

Copy and paste relevant sections from the manuscript (include quotes in quotation marks "like this" to indicate direct quotes from your manuscript), or elaborate on this item by providing additional information not in the ms, or briefly explain why the item is not applicable/relevant for your study

"Linear mixed models in SPSS version 25 (IBM Corp, Armonk, NY, USA) were used to conduct intent-to-treat analyses, without prediction of missing data, on the primary outcome variable of difficulties in emotion regulation and secondary outcomes of mental distress and well-being. "

12b) Methods for additional analyses, such as subgroup analyses and adjusted analyses

Does your paper address CONSORT subitem 12b? *

Copy and paste relevant sections from the manuscript (include quotes in quotation marks "like this" to indicate direct quotes from your manuscript), or elaborate on this item by providing additional information not in the ms, or briefly explain why the item is not applicable/relevant for your study

N/A

X26) REB/IRB Approval and Ethical Considerations [recommended as subheading under "Methods"] (not a CONSORT item)

X26-i) Comment on ethics committee approval

	1	2	3	4	5	
subitem not at all important	○	○	●	○	○	essential

Does your paper address subitem X26-i?

Copy and paste relevant sections from the manuscript (include quotes in quotation marks "like this" to indicate direct quotes from your manuscript), or elaborate on this item by providing additional information not in the ms, or briefly explain why the item is not applicable/relevant for your study

"Ethical approval was granted by the relevant university human research ethics committees"

x26-ii) Outline informed consent procedures

Outline informed consent procedures e.g., if consent was obtained offline or online (how? Checkbox, etc.?), and what information was provided (see 4a-ii). See [6] for some items to be included in informed consent documents.

	1	2	3	4	5	
subitem not at all important	◉	○	○	○	○	essential

Does your paper address subitem X26-ii?

Copy and paste relevant sections from the manuscript (include quotes in quotation marks "like this" to indicate direct quotes from your manuscript), or elaborate on this item by providing additional information not in the ms, or briefly explain why the item is not applicable/relevant for your study

"consent was obtained online before participants completed the baseline online survey" by clicking a checkbox.

X26-iii) Safety and security procedures

Safety and security procedures, incl. privacy considerations, and any steps taken to reduce the likelihood or detection of harm (e.g., education and training, availability of a hotline)

	1	2	3	4	5	
subitem not at all important	○	◉	○	○	○	essential

Does your paper address subitem X26-iii?

Copy and paste relevant sections from the manuscript (include quotes in quotation marks "like this" to indicate direct quotes from your manuscript), or elaborate on this item by providing additional information not in the ms, or briefly explain why the item is not applicable/relevant for your study

N/A

RESULTS

13a) For each group, the numbers of participants who were randomly assigned, received intended treatment, and were analysed for the primary outcome

NPT: The number of care providers or centers performing the intervention in each group and the number of patients treated by each care provider in each center

Does your paper address CONSORT subitem 13a? *

Copy and paste relevant sections from the manuscript (include quotes in quotation marks "like this" to indicate direct quotes from your manuscript), or elaborate on this item by providing additional information not in the ms, or briefly explain why the item is not applicable/relevant for your study

"Figure 2 displays the consort diagram. A total of 209 young people responded to recruitment advertisements and completed the online survey. Of those, 80.9% (169/209) met full study inclusion criteria and were allocated to immediate (n=85) or delayed (n=84) app access."

13b) For each group, losses and exclusions after randomisation, together with reasons

Does your paper address CONSORT subitem 13b? (NOTE: Preferably, this is shown in a CONSORT flow diagram) *

Copy and paste relevant sections from the manuscript (include quotes in quotation marks "like this" to indicate direct quotes from your manuscript), or elaborate on this item by providing additional information not in the ms, or briefly explain why the item is not applicable/relevant for your study

"Follow-up rates were high (93.5% at 1 month, 87.6% at 2 months, 88.2% at 3 months, and 84.0% at 6 months)"

13b-i) Attrition diagram

Strongly recommended: An attrition diagram (e.g., proportion of participants still logging in or using the intervention/comparator in each group plotted over time, similar to a survival curve) or other figures or tables demonstrating usage/dose/engagement.

	1	2	3	4	5	
subitem not at all important	○	○	○	○	◉	essential

Does your paper address subitem 13b-i?

Copy and paste relevant sections from the manuscript or cite the figure number if applicable (include quotes in quotation marks "like this" to indicate direct quotes from your manuscript), or elaborate on this item by providing additional information not in the ms, or briefly explain why the item is not applicable/relevant for your study

CONSORT diagram provided in manuscript: "Figure 2"

14a) Dates defining the periods of recruitment and follow-up

Does your paper address CONSORT subitem 14a? *

Copy and paste relevant sections from the manuscript (include quotes in quotation marks "like this" to indicate direct quotes from your manuscript), or elaborate on this item by providing additional information not in the ms, or briefly explain why the item is not applicable/relevant for your study

Recruitment for this study was done October, 2014 - March, 2015

14a-i) Indicate if critical "secular events" fell into the study period

Indicate if critical "secular events" fell into the study period, e.g., significant changes in Internet resources available or "changes in computer hardware or Internet delivery resources"

	1	2	3	4	5	
subitem not at all important	◉	○	○	○	○	essential

Does your paper address subitem 14a-i?

Copy and paste relevant sections from the manuscript (include quotes in quotation marks "like this" to indicate direct quotes from your manuscript), or elaborate on this item by providing additional information not in the ms, or briefly explain why the item is not applicable/relevant for your study

N/A

14b) Why the trial ended or was stopped (early)

Does your paper address CONSORT subitem 14b? *

Copy and paste relevant sections from the manuscript (include quotes in quotation marks "like this" to indicate direct quotes from your manuscript), or elaborate on this item by providing additional information not in the ms, or briefly explain why the item is not applicable/relevant for your study

N/A

15) A table showing baseline demographic and clinical characteristics for each group

NPT: When applicable, a description of care providers (case volume, qualification, expertise, etc.) and centers (volume) in each group

Does your paper address CONSORT subitem 15? *

Copy and paste relevant sections from the manuscript (include quotes in quotation marks "like this" to indicate direct quotes from your manuscript), or elaborate on this item by providing additional information not in the ms, or briefly explain why the item is not applicable/relevant for your study

"Demographic characteristics of the sample are displayed in Table 1"

15-i) Report demographics associated with digital divide issues

In ehealth trials it is particularly important to report demographics associated with digital divide issues, such as age, education, gender, social-economic status, computer/Internet/ehealth literacy of the participants, if known.

	1	2	3	4	5	
subitem not at all important	○	○	○	○	◉	essential

Does your paper address subitem 15-i? *

Copy and paste relevant sections from the manuscript (include quotes in quotation marks "like this" to indicate direct quotes from your manuscript), or elaborate on this item by providing additional information not in the ms, or briefly explain why the item is not applicable/relevant for your study

The following demographics have been reported: "Age, mean (SD)
Gender (females), n (%)
English language fluency, n (%)
Aboriginal or Torres Strait Islander, n (%)
Education, n (%)
Degree, postgraduate study
Certificate or diploma
High school
Work status, n (%)
Full-time
Part-time or casual
Unemployed or disability allowance
Home duties
Volunteer work
Full-time or part-time student
Relationship status, n (%)
Married
In a relationship
Single
Current psychological treatment, n (%)
Use smartphone daily, n (%)
Music, n (%)
Accessed music online in past month
Play musical instrument or sing in choir
  Past
  Current
Compose music
Attend concerts or live music at least monthly
Music listening each week, mean (SD)
  Average number of hours per day
  Average number of days
  Median split (days×hours)—High
  HUMS*
    High healthy music use
    High unhealthy music use
At least moderate success at listening to music to change mood, n (%)

*HUMS: Healthy Unhealthy Music scale."

16) For each group, number of participants (denominator) included in each analysis and whether the analysis was by original assigned groups

16-i) Report multiple “denominators” and provide definitions

Report multiple “denominators” and provide definitions: Report N’s (and effect sizes) “across a range of study participation [and use] thresholds” [1], e.g., N exposed, N consented, N used more than x times, N used more than y weeks, N participants “used” the intervention/comparator at specific pre-defined time points of interest (in absolute and relative numbers per group). Always clearly define “use” of the intervention.

	1	2	3	4	5	
subitem not at all important	○	○	○	○	◉	essential

Does your paper address subitem 16-i? *

Copy and paste relevant sections from the manuscript (include quotes in quotation marks "like this" to indicate direct quotes from your manuscript), or elaborate on this item by providing additional information not in the ms, or briefly explain why the item is not applicable/relevant for your study

"immediate (n=85) or delayed (n=84) app access"
"Backend data indicated that 12 participants did not download the app, a further 34 downloaded but never used the app, 31 downloaded it but experienced technical flaws, and 7 were allocated to the immediate condition but did not download the app until a month after allocation (included in this group for intent-to-treat purposes)."

16-ii) Primary analysis should be intent-to-treat

Primary analysis should be intent-to-treat, secondary analyses could include comparing only “users”, with the appropriate caveats that this is no longer a randomized sample (see 18-i).

	1	2	3	4	5	
subitem not at all important	○	○	○	○	◉	essential

Does your paper address subitem 16-ii?

Copy and paste relevant sections from the manuscript (include quotes in quotation marks "like this" to indicate direct quotes from your manuscript), or elaborate on this item by providing additional information not in the ms, or briefly explain why the item is not applicable/relevant for your study

""Linear mixed models ... were used to conduct intent-to-treat analyses, without prediction of missing data"

17a) For each primary and secondary outcome, results for each group, and the estimated effect size and its precision (such as 95% confidence interval)

Does your paper address CONSORT subitem 17a? *

Copy and paste relevant sections from the manuscript (include quotes in quotation marks "like this" to indicate direct quotes from your manuscript), or elaborate on this item by providing additional information not in the ms, or briefly explain why the item is not applicable/relevant for your study

The linear mixed model revealed no time by group interaction for any of the 6 difficulties in emotion regulation subscales of the DERS (see Table 3). Time effects were found on 5 of the 6 DERS subscales (clarity, goals, nonacceptance, strategies, and impulse) when comparing baseline both with the 1-month follow-up and with the 2-, 3-, and 6-month follow-ups (see Table 3). These effects did not vary when controlling for whether participants used the app (yes or no) or the level of app use.

To better understand these changes over time, moderating effects of gender, duration of music use, and healthy or unhealthy music use were assessed across all time points. For difficulties engaging in goal-directed behavior when distressed (DERS-Goal) and nonacceptance of emotional responses (DERS-nonacceptance), no significant moderating effects were found for gender ($F_{4,328}$=2.12; P=.07; $F_{4,351}$=1.16; P=.33), duration of music use ($F_{4,361}$=1.00; p=.40; $F_{4,366}$=0.32; p=.87), unhealthy use of music ($F_{4,369}$=0.53; p=.72; $F_{4,373}$=1.98; p=.09), or healthy use of music ($F_{4,363}$=1.63; p=.17; $F_{4,355}$=0.20; p=.94). When exploring the time main effects, difficulties engaging in goal-directed behavior decreased from baseline to 1 month (meandifference=−1.04, 95% CI −1.44 to −0.65; t_{383}=5.24; p<.001; d=0.36) and from 1 to 2 months (meandifference=−0.59, 95% CI −1.03 to −0.14; t_{383}=2.59; p=.01; d=0.22), before maintaining stability at 3 and 6 months (p=.72; p=.80; see Figure 3).

Nonacceptance of emotional responses decreased from baseline to 1 month (meandifference=−0.59, 95% CI −1.41 to −0.55; t_{376}=4.49; p<.001; d=0.32) and maintained stability thereafter (p=.46; p=.81; p=.82).

17a-i) Presentation of process outcomes such as metrics of use and intensity of use

In addition to primary/secondary (clinical) outcomes, the presentation of process outcomes such as metrics of use and intensity of use (dose, exposure) and their operational definitions is critical. This does not only refer to metrics of attrition (13-b) (often a binary variable), but also to more continuous exposure metrics such as “average session length”. These must be accompanied by a technical description how a metric like a “session” is defined (e.g., timeout after idle time) [1] (report under item 6a).

	1	2	3	4	5	
subitem not at all important	○	○	○	○	◉	essential

Does your paper address subitem 17a-i?

Copy and paste relevant sections from the manuscript (include quotes in quotation marks "like this" to indicate direct quotes from your manuscript), or elaborate on this item by providing additional information not in the ms, or briefly explain why the item is not applicable/relevant for your study

"Backend data indicated that 12 participants did not download the app, a further 34 downloaded but never used the app, 31 downloaded it but experienced technical flaws, and 7 were allocated to the immediate condition but did not download the app until a month after allocation (included in this group for intent-to-treat purposes). Of those who downloaded the app, the total number of generated playlists ranged from 0 to 71, (median=2). No playlists were generated by 41%, and only 7.5% of the sample generated more than 15 playlists. The number of generated playlists did not vary significantly between immediate- and delayed-access groups or by gender. The duration of app music use variable was considered unreliable as it was not possible to gauge the extent to which participants were listening to the music (vs leaving the app open with music playing)."

17b) For binary outcomes, presentation of both absolute and relative effect sizes is recommended

Does your paper address CONSORT subitem 17b? *

Copy and paste relevant sections from the manuscript (include quotes in quotation marks "like this" to indicate direct quotes from your manuscript), or elaborate on this item by providing additional information not in the ms, or briefly explain why the item is not applicable/relevant for your study

N/A

18) Results of any other analyses performed, including subgroup analyses and adjusted analyses, distinguishing pre-specified from exploratory

Does your paper address CONSORT subitem 18? *

Copy and paste relevant sections from the manuscript (include quotes in quotation marks "like this" to indicate direct quotes from your manuscript), or elaborate on this item by providing additional information not in the ms, or briefly explain why the item is not applicable/relevant for your study

On the uMARS, the app had a high level of objective app quality (meanoverall=3.8 [SD 0.50]), with good engagement (mean 3.67 [SD 0.61]), aesthetics (mean 4.10 [SD 0.63]), and information (mean 4.05 [SD 0.61]), and acceptable functionality (mean 3.47 [SD 0.66]). Participants reported they would use the app between 10 and 50 times (mean 4.09 [SD 1.04]), and although they were unlikely to pay for the app (mean 2.43 [SD 1.23]), they gave it a 3.6 out of 5-star rating (SD 0.65).

18-i) Subgroup analysis of comparing only users

A subgroup analysis of comparing only users is not uncommon in ehealth trials, but if done, it must be stressed that this is a self-selected sample and no longer an unbiased sample from a randomized trial (see 16-iii).

	1	2	3	4	5	
subitem not at all important	(●)	( )	( )	( )	( )	essential

Does your paper address subitem 18-i?

Copy and paste relevant sections from the manuscript (include quotes in quotation marks "like this" to indicate direct quotes from your manuscript), or elaborate on this item by providing additional information not in the ms, or briefly explain why the item is not applicable/relevant for your study

N/A

19) All important harms or unintended effects in each group

(for specific guidance see CONSORT for harms)

Does your paper address CONSORT subitem 19? *

Copy and paste relevant sections from the manuscript (include quotes in quotation marks "like this" to indicate direct quotes from your manuscript), or elaborate on this item by providing additional information not in the ms, or briefly explain why the item is not applicable/relevant for your study

N/A

19-i) Include privacy breaches, technical problems

Include privacy breaches, technical problems. This does not only include physical “harm” to participants, but also incidents such as perceived or real privacy breaches [1], technical problems, and other unexpected/unintended incidents. “Unintended effects” also includes unintended positive effects [2].

	1	2	3	4	5	
subitem not at all important	○	○	◉	○	○	essential

Does your paper address subitem 19-i?

Copy and paste relevant sections from the manuscript (include quotes in quotation marks "like this" to indicate direct quotes from your manuscript), or elaborate on this item by providing additional information not in the ms, or briefly explain why the item is not applicable/relevant for your study

"31 downloaded it but experienced technical flaws, and 7 were allocated to the immediate condition but did not download the app until a month after allocation (included in this group for intent-to-treat purposes)"

19-ii) Include qualitative feedback from participants or observations from staff/researchers

Include qualitative feedback from participants or observations from staff/researchers, if available, on strengths and shortcomings of the application, especially if they point to unintended/unexpected effects or uses. This includes (if available) reasons for why people did or did not use the application as intended by the developers.

	1	2	3	4	5	
subitem not at all important	◉	○	○	○	○	essential

Does your paper address subitem 19-ii?

Copy and paste relevant sections from the manuscript (include quotes in quotation marks "like this" to indicate direct quotes from your manuscript), or elaborate on this item by providing additional information not in the ms, or briefly explain why the item is not applicable/relevant for your study

N/A

DISCUSSION

22) Interpretation consistent with results, balancing benefits and harms, and considering other relevant evidence

NPT: In addition, take into account the choice of the comparator, lack of or partial blinding, and unequal expertise of care providers or centers in each group

22-i) Restate study questions and summarize the answers suggested by the data, starting with primary outcomes and process outcomes (use)

Restate study questions and summarize the answers suggested by the data, starting with primary outcomes and process outcomes (use).

	1	2	3	4	5	
subitem not at all important	○	○	○	○	●	essential

Does your paper address subitem 22-i? *

Copy and paste relevant sections from the manuscript (include quotes in quotation marks "like this" to indicate direct quotes from your manuscript), or elaborate on this item by providing additional information not in the ms, or briefly explain why the item is not applicable/relevant for your study

"This study examined the 1-month efficacy and 2-, 3-, and 6-month outcomes of the Music eScape app in 169 young people with at least mild levels of mental distress. The trial found no differential improvements from app access at 1 month in emotion regulation, mental distress, or well-being. Nevertheless, improvements on 5 out of the 6 emotion regulation strategies, mental distress, and well-being were evident in both groups over the 6-month trial.

The lack of significant differences between the immediate versus 1-month delayed-access groups indicates that the Music eScape app was ineffective in achieving change in emotion regulation, mental distress, or well-being, beyond the impact of research assessments alone. Gender, duration of music use, unhealthy and healthy music use, and app use did not impact these results. Although the use of a 1-month delayed-access control may have limited our ability to find effects, waitlist control conditions are commonly used in mobile health (mHealth) research. For example, 2 recent meta-analyses of smartphone mental health apps reporting 8 out of 18 studies on depression and 4 out of 9 studies on anxiety used waitlist controls [61,62]. The duration of the delay ranged from 4 to 16 weeks, depending on the length of the mHealth intervention [61,62]. Although the meta-analyses found mHealth apps had small to moderate effects on both depression and anxiety outcomes, 2 out of the 3 included studies that used a 1-month waitlist control found no effects [56,57]. Thus, the 1-month delay used in this trial might have been insufficient for participants to receive an adequate dose of the Music eScape app. Baseline data also indicated that participants had high levels of music use (2.6 hours per day) and emotional awareness (DERS subscale), and 88% participants reported at least moderate levels of success using music to change their mood, suggesting a ceiling effect may have been present on these variables. Nevertheless, improvements in emotion regulation were found on the 5 DERS subscales across the whole sample, suggesting that this study had the ability to detect a change in emotion regulation across time.

Both groups had access to the Music eScape app after the first month. Although improvements in mental distress, well-being, and emotion regulation were found over the 6 months, it is not possible to attribute these results to the app. These improvements may have been because of regression to the mean or assessment effects, particularly given that the 5 assessments were completed over a 6-month period. The amount of app use did not affect any outcomes.

To better understand the changes in emotion regulation strategies, over time, moderating effects of gender, duration of music use, and healthy or unhealthy music use were explored. Results indicated that improvements in emotional clarity, impulse control, and limited access to emotion regulation strategies were

only found in distressed young people who engaged in high levels of unhealthy music use at baseline. This finding highlights the potential importance of targeting unhealthy music use to improve the emotion regulation skills of distressed young people. Currently, the app allows users to maintain or intensify their current mood by choosing mood-congruent music. The moderation of outcomes by the degree of unhealthy use of music suggests that some may have used the app to stay in a negative mood, consistent with observations in previous research [30-32]. According to meta-emotion theories, some people are drawn toward emotional experiences, whereas others are motivated to avoid and control emotional experiences. Bartsch et al [44] draw links between these individual meta-emotional tendencies and selective of media use such as watching melodramatic or horror movies (which some people enjoy, whereas others prefer to avoid). Similarly, some people enjoy listening to sad and angry music [63], and there is some evidence that this music-induced emotional exposure is related to better emotional processing and well-being [64-66]. Other research suggests that immersion in sad and angry music might be unhealthy for some young people who are prone to depression or other mental health problems [37]. Further research is required to determine whether the Music eScape app can improve emotion regulation skills through reductions in unhealthy music use, particularly if coaching is provided on its use for that purpose. Prospective research is also required to determine whether unhealthy music use is a correlate or risk factor for depression in young people and whether it moderates emotion regulation skills in young people without depression.
No moderators of well-being outcomes across the 6 months were found, and only female gender, but not the amount or type (healthy or unhealthy) of music use, moderated improvements in mental distress. The reduction in distress for female but not male participants may be partly because of increased power in detecting changes for females, given that 79% of the sample was female. However, no gender differences in app use were found, and gender has not been found to moderate depression or anxiety outcomes in systematic reviews of psychological treatment trials [67,68]. "

22-ii) Highlight unanswered new questions, suggest future research

Highlight unanswered new questions, suggest future research.

	1	2	3	4	5	
subitem not at all important	○	○	○	○	◉	essential

Does your paper address subitem 22-ii?

Copy and paste relevant sections from the manuscript (include quotes in quotation marks "like this" to indicate direct quotes from your manuscript), or elaborate on this item by providing additional information not in the ms, or briefly explain why the item is not applicable/relevant for your study

"Further research is required to determine if gender moderates eHealth treatment outcomes."
"Although further testing is required to demonstrate efficacy, the results of this study highlight the potential of music interventions apps such as Music eScape to deliver engaging and highly accessible emotion regulation skills training to young people in real time in their natural environment, which in principle could result in population-wide benefits in mental distress and well-being. "
"However, there might have been other unmeasured moderators that future research may identify."
"The music available through the current version of Music eScape is limited to the users' own music library. However, 97% of the sample reported accessing music online in the past week. Future versions of the app, which interface with music streaming services that give users access to a much wider repertoire, may enhance its effects by giving users a choice of preferred music for each mood journey step or ensuring that different (or more current) music is offered each time they use the app.
Further testing is required to demonstrate whether the app has effects on emotion regulation, mental health, and well-being over a longer delayed-access period, and if so, whether it has superior effects compared with placebo control apps or other emotion regulation apps or interventions. Additional benefits from adding the app to other interventions for emotion regulation in young people could also be tested."

20) Trial limitations, addressing sources of potential bias, imprecision, and, if relevant, multiplicity of analyses

20-i) Typical limitations in ehealth trials

Typical limitations in ehealth trials: Participants in ehealth trials are rarely blinded. Ehealth trials often look at a multiplicity of outcomes, increasing risk for a Type I error. Discuss biases due to non-use of the intervention/usability issues, biases through informed consent procedures, unexpected events.

	1	2	3	4	5	
subitem not at all important	○	○	○	○	◉	essential

Does your paper address subitem 20-i? *

Copy and paste relevant sections from the manuscript (include quotes in quotation marks "like this" to indicate direct quotes from your manuscript), or elaborate on this item by providing additional information not in the ms, or briefly explain why the item is not applicable/relevant for your study

"A large community sample of 169 young people with at least mild distress (K10 scores of >17) participated in this trial. However, the volunteer sampling method used to recruit participants limits the generalizability of results. Despite efforts to avoid participants with an affinity for music during recruitment, the sample used music for substantial average durations at baseline (2+ hours per 6 days a week). Many also used music for emotion regulation strategy, which may have created ceiling effects on key outcome variables. We were also only able to use the number of app playlists generated as an indicator of app use rather than the duration of app music use."

21) Generalisability (external validity, applicability) of the trial findings

NPT: External validity of the trial findings according to the intervention, comparators, patients, and care providers or centers involved in the trial

21-i) Generalizability to other populations

Generalizability to other populations: In particular, discuss generalizability to a general Internet population, outside of a RCT setting, and general patient population, including applicability of the study results for other organizations

	1	2	3	4	5	
subitem not at all important	○	○	●	○	○	essential

Does your paper address subitem 21-i?

Copy and paste relevant sections from the manuscript (include quotes in quotation marks "like this" to indicate direct quotes from your manuscript), or elaborate on this item by providing additional information not in the ms, or briefly explain why the item is not applicable/relevant for your study

"the volunteer sampling method used to recruit participants limits the generalizability of results"

21-ii) Discuss if there were elements in the RCT that would be different in a routine application setting

Discuss if there were elements in the RCT that would be different in a routine application setting (e.g., prompts/reminders, more human involvement, training sessions or other co-interventions) and what impact the omission of these elements could have on use, adoption, or outcomes if the intervention is applied outside of a RCT setting.

	1	2	3	4	5	
subitem not at all important	(●)	○	○	○	○	essential

Does your paper address subitem 21-ii?

Copy and paste relevant sections from the manuscript (include quotes in quotation marks "like this" to indicate direct quotes from your manuscript), or elaborate on this item by providing additional information not in the ms, or briefly explain why the item is not applicable/relevant for your study

Your answer

OTHER INFORMATION

23) Registration number and name of trial registry

Does your paper address CONSORT subitem 23? *

Copy and paste relevant sections from the manuscript (include quotes in quotation marks "like this" to indicate direct quotes from your manuscript), or elaborate on this item by providing additional information not in the ms, or briefly explain why the item is not applicable/relevant for your study

Australian New Zealand Clinical Trials Registry (ACTRN12615000051549)

24) Where the full trial protocol can be accessed, if available

Does your paper address CONSORT subitem 24? *

Cite a Multimedia Appendix, other reference, or copy and paste relevant sections from the manuscript (include quotes in quotation marks "like this" to indicate direct quotes from your manuscript), or elaborate on this item by providing additional information not in the ms, or briefly explain why the item is not applicable/relevant for your study

http://www.anzctr.org.au/

25) Sources of funding and other support (such as supply of drugs), role of funders

Does your paper address CONSORT subitem 25? *

Copy and paste relevant sections from the manuscript (include quotes in quotation marks "like this" to indicate direct quotes from your manuscript), or elaborate on this item by providing additional information not in the ms, or briefly explain why the item is not applicable/relevant for your study

"The Young and Well Cooperative Research Centre (Young and Well CRC; 2011-2016) funded this project. The Young and Well CRC was an Australian-based international research center established under the Australian Government's Cooperative Research Centres Program."

X27) Conflicts of Interest (not a CONSORT item)

X27-i) State the relation of the study team towards the system being evaluated

In addition to the usual declaration of interests (financial or otherwise), also state the relation of the study team towards the system being evaluated, i.e., state if the authors/evaluators are distinct from or identical with the developers/sponsors of the intervention.

	1	2	3	4	5	
subitem not at all important	○	○	◉	○	○	essential

Does your paper address subitem X27-i?

Copy and paste relevant sections from the manuscript (include quotes in quotation marks "like this" to indicate direct quotes from your manuscript), or elaborate on this item by providing additional information not in the ms, or briefly explain why the item is not applicable/relevant for your study

Authors are identical to the developers.
"funders had no role in the study design; collection, analysis, or interpretation of data; writing the manuscript; and the decision to submit the manuscript for publication."

About the CONSORT EHEALTH checklist

As a result of using this checklist, did you make changes in your manuscript? *

- ○ yes, major changes
- ○ yes, minor changes
- ◉ no

What were the most important changes you made as a result of using this checklist?

N/A

How much time did you spend on going through the checklist INCLUDING making changes in your manuscript *

10 hours

As a result of using this checklist, do you think your manuscript has improved? *

- ○ yes
- ◉ no
- ○ Other:

Would you like to become involved in the CONSORT EHEALTH group?

This would involve for example becoming involved in participating in a workshop and writing an "Explanation and Elaboration" document

- ○ yes
- ◉ no
- ○ Other:

Any other comments or questions on CONSORT EHEALTH

Your answer

STOP - Save this form as PDF before you click submit

To generate a record that you filled in this form, we recommend to generate a PDF of this page (on a Mac, simply select "print" and then select "print as PDF") before you submit it.

When you submit your (revised) paper to JMIR, please upload the PDF as supplementary file.

Don't worry if some text in the textboxes is cut off, as we still have the complete information in our database. Thank you!

Final step: Click submit !

Click submit so we have your answers in our database!

SUBMIT

Never submit passwords through Google Forms.

This content is neither created nor endorsed by Google. Report Abuse - Terms of Service

Google Forms
